# Evaluation of pharmaceutical care services in the Middle East Countries: a review of studies of 2013–2020

**DOI:** 10.1186/s12889-023-16199-1

**Published:** 2023-07-17

**Authors:** Hebah Sallom, Abdikarim Abdi, Abdulsalam M Halboup, Bilgen Başgut

**Affiliations:** 1 Department of Clinical Pharmacy, Faculty of Pharmacy, Near East University, Nicosia, Cyprus, Turkey; 2 Department of Clinical Pharmacy and Practice, Faculty of Pharmacy, University of Science and Technology, Sana’a, Yemen; 3 Department of Clinical Pharmacy, Faculty of Pharmacy, Yeditepe University, İstanbul, Turkey; 4 Department of Clinical Pharmacy and Practice, Faculty of Pharmacy, University of Science and Technology, Sana’a, Yemen; 5 Discipline of Clinical Pharmacy, School of Pharmaceutical Sciences, Universiti Sains Malaysia, Penang, Malaysia; 6 Department of Pharmacology, Faculty of Pharmacy, Başkent University, Ankara, Turkey

**Keywords:** Assessment, Clinical pharmacy services, Middle East, Pharmaceutical Care Services, Pharmacy practice

## Abstract

**Introduction:**

Pharmaceutical care services (PCs) have evolved significantly over the last few decades, with a greater focus on patient’s safety and proven effectiveness in a wide range of contexts. Many of the evidence supporting this technique comes from the United States, the evaluation and adoption of (PCs) which differ greatly across the globe.

**Objective:**

The goal of this study was to identify and assess the efficacy of pharmaceutical care services in various pharmaceutical aspects throughout seventeen Middle Eastern nations.

**Method:**

The Arkesy and O’Malley technique was used to conduct a scoping review. It was conducted using PubMed/Medline, Scopus, Cochrane Library, Springer Link, Clinical Trials, and Web of Science etc. The Van Tulder Scale was utilized in randomized trials research, whereas the dawn and black checklists were used in non-randomized trials research. A descriptive and numerical analysis of selected research was done. The scope of eligible PCs, pharmaceutical implementers, study outcomes, and quality were all identified by a thematic review of research.

**Results:**

There were about 431,753 citations found in this study, and 129 publications were found to be eligible for inclusion after analysing more than 271 full-text papers. The study design was varied, with 43 (33.3%) RCTs and 86 (66.7%) n-RCTs. Thirty-three (25.6%) of the studies were published in 2020. Jordan, Saudi Arabia, and Turkey were home to the majority of the studies (25.6%, 16.3%, and 11.6%) respectively. Thirty-seven studies (19.7%) were concerned with resolving drug related problems (DRPs), whereas 27 (14.4%) were concerned with increasing quality of life (QOL) and 23 (12.2%) with improving drug adherence. Additionally, the research revealed that the average ratings of the activities provided to patients improved every year.

**Conclusion:**

Studies in the Middle East continue to provide evidence supporting the positive impact of pharmaceutical care services on both hard and soft outcomes measured in most studies. Yet there was rare focus on the value of the implemented services. Thus, rigorous evaluation of the economic impact of implemented pharmaceutical care services in the Middle East and assessment of their sustainability is must.

**Supplementary Information:**

The online version contains supplementary material available at 10.1186/s12889-023-16199-1.

## Introduction

Despite the fact that pharmaceutical care (PC) has evolved into a whole philosophy and a standard for providing health care as a result of pharmacy organizations’ continuing professional development programs around the world [1], the importance of pharmaceutical care services and the implementation of pharmacy practice research has not been clearly defined in the Middle East countries so that independent observers can detect its presence, strength, and sustainability [2].

In general, because of disparities in training programs, healthcare systems, and regulatory frameworks, the state of pharmacy practice in the Middle Eastern differs among various nations. However, because of the near vicinity of the two locations, the same language, culture, and history, there are many parallels in terms of practicing facilitators and barriers [3].

A more modern definition of Pharmaceutical Care Network Europe (PCNE), which specifies pharmaceutical care as a service provided by pharmacists, states: Pharmaceutical Care is that the pharmacist’s contribution to the care of people is to optimize the use of medicines and improve health outcomes [4].

The pharmacist’s roles in patient care have expanded from the traditional tasks of dispensing medications and providing basic medication counseling to working with other health care professionals and the general community. This has resulted greater in the pharmacist’s participation in different health care systems involving in/out-patients pharmacies, general medical practices, and hospitals [5]. Six papers from Jordan, the United Arab Emirates, Sudan, and Iraq were found in a systematic review looking at pharmacists’ intervention programs for diabetic patients in these Arab nations. These trials revealed considerable gains in patients’ knowledge, adherence, and therapeutic results [6].

On the other hand, another systematic review looked at how people felt about the role and offerings of community pharmacists in Arabic-speaking Middle Eastern nations, showing the widespread belief that these professionals offer services that are business-oriented. It was also observed that the public’s impression of the pharmacist’s involvement in clinically oriented services was poor [3].

The pharmacy profession has been thriving in the Middle East. In Jordan, we found that the number of community pharmacies has surpassed 2200, and the active workforce is anticipated to include 12,000 registered pharmacists. In Jordan, pharmaceutical care services are highly developed in recent years, resulting in improved patient outcomes and therapy management in different medical conditions and healthcare settings [7–10].

Another systematic review analyzed suggestions for program creation and looked at how antimicrobial stewardship methods affected prescribing practices and antibiotic appropriateness in the Middle East. Giving clinical pharmacists a more prominent role in the administration and prescription of antibiotics was one of the recommendations made by Jordan, the United Arab Emirates, Qatar, Saudi Arabia, and Lebanon [11].

Although the benefits of pharmaceutical care interventions by pharmacists within various healthcare settings have been documented in the literature, particularly in systematic reviews, [12–17] there is a need to evaluate the impact holistically through a multidimensional assessment of clinical, economic, and humanistic outcomes [18].

Identifying these issues can help in determining what factors impede or facilitate intervention, the development of solutions to overcome process obstacles, the introduction of innovations into health systems, or the promotion of their widespread usage and sustainability [19]. Implementation research may be a crucial method for ensuring the successful adoption and sustainability of Clinical Pharmacy Services (CPS) in countries where they have not yet been established [20–22].

However, Pew Health Profession Commission (PHPC) recommended that pharmaceutical education “should begin with a curricular reform to be qualified to perform pharmaceutical care” [23]. By looking at the educational side of the Middle East region, some studies showed that some of the universities who nationally certified for pharmacy schools have international accreditation from recognized pharmacy authorities [24].

According to a study of the current state of PCs services in a number of Arabic-speaking Middle East (ME) countries, the pharmaceutical care concept has only recently been introduced to many (ME) countries, and is still frequently confused with clinical pharmacy, which remains a priority in several countries of the region. In many Middle Eastern nations, pharmacy education is undergoing rapid transition and some pharmacy institutions have launched or plan to introduce the Pharm.D degree to replace the traditional Bachelor of Pharmaceutical Science degree. These improvements are expected to reflect a greater understanding and implementation of pharmaceutical care in various forms, such as medication management services, in hospital and community settings [25].

These services will not only help in determining and approving the critical tasks of pharmacists or clinical pharmacists aid or improving the implementation of Pharmaceutical Care services, but will also increase collaboration between pharmacists and other health care providers. Therefore, the purpose of this search investigation was to answer the following research question:

1. Does pharmaceutical Care Services optimize rational use of medicine?
2. Does pharmaceutical Care Services promote health and well- being?
3. Does pharmaceutical Care Services improve patient’s therapeutic outcome?
4. Does pharmaceutical Care Services help in the prevention of diseases?

## Materials and methods

### Information sources and search strategy

A complete literature review was conducted in the following databases between 2013 and 2020: PubMed/Medline, Scopus, Cochrane Library, Springer Link, Clinical Trial, and Web of Science. The phases of the Arkesy and O’Malley framework we used to perform a scoping study were as follows:

Phase No.1: Identifying the research question

Phase No.2: Identifying relevant studies

Phase No.3: Study selection

Phase No.4: charting the data

Phase No.5: Summarizing and reporting results

A specific words were used included in: ((“Clinical pharmacy practice” OR “Pharmacy practice” OR “Pharmaceutical Care” OR Pharmacy OR Pharmacist) AND (Service OR Intervention OR Program OR Evaluation OR Assessment OR indicators OR Quality) AND (“Middle East Countries” OR “Low Income Countries” OR “Developed countries” OR “under developed countries” OR “Developing countries” OR Bahrain OR Cyprus OR Egypt OR Iran OR Iraq OR Israel OR Jordan OR Kuwait OR Lebanon OR Oman OR Palestine OR Qatar OR “Saudi Arabia” OR Syria OR Turkey OR “The United Arab Emirates” OR Yemen)).All terms in each database incorporated with Boolean operators (AND, OR and/or NOT).

### Study inclusion

Studies that evaluated different pharmaceutical care services provided by pharmacists or clinical pharmacists for hospitalized patients or out-patients, full-text journals, articles written in English only, and studies conducted only in Middle Eastern countries, such as Bahrain, Cyprus, Egypt, Iran, Iraq, Israel, Jordan, Kuwait, Lebanon, Oman, Palestine, Qatar, Saudi Arabia, Syria, Turkey, United Arab Emirates, and Yemen, were included in this review. Selection of these countries based on the following portals: WORLD BANK portal (https://data.worldbank.org/region/least-developed-countries-un-classification) and World Population Review (https://worldpopulationreview.com/country-rankings/middle-east-countries). Studies that were a summary of the literature for the purpose of gathering information or opinion, an editorial, a discussion paper, or a meeting abstract were all removed.

### Data collection and analysis

**Fig. 1:**
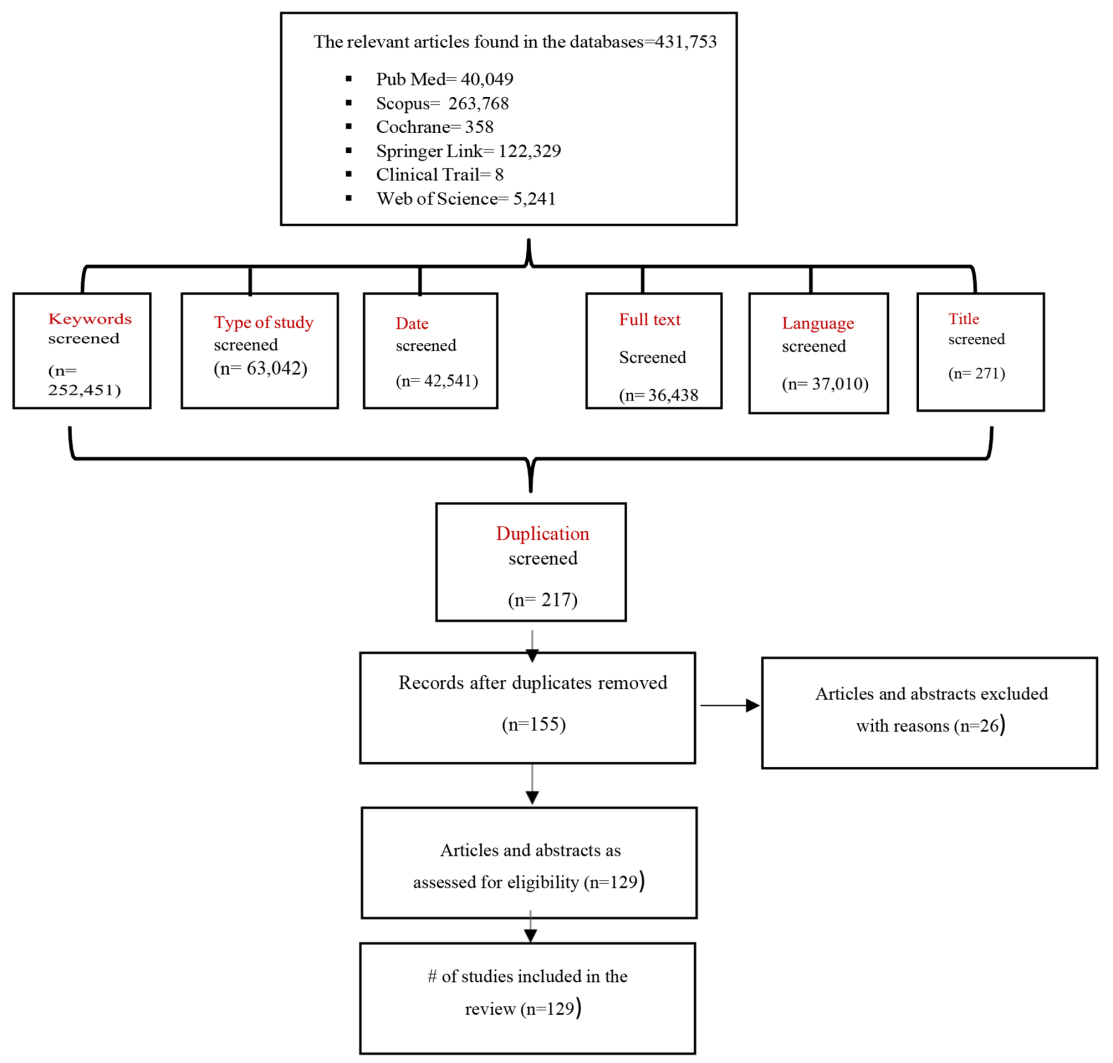
Flow diagram describes the procedures involved in the search and selection process for Randomized and Non-Randomized controlled studies

Once we completed the searching on electronic databases, we conducted a hand- search and scanned the reference lists from relevant papers to identify other papers that may not have been found in the initial search. Then, all the initially determined studies were analyzed by two reviewers their titles and abstracts based on the selection criteria’s Fig. 1. Then two reviewer authors independently screened for the full text of the first phase of study selection of the eligible studies for their second stage of screening and any disagreements about studies selection were discussed and resolved. These eligible studies were uploaded into Microsoft Excel Software and reviewed all data extracted in the sheets.

The data extracted included: Title, Year of publication, Aim/s, Location, Language and Type of study. These studies were sorted into Randomized and Non-Randomized studies to start their quality evaluation. Statistical Package for the Social Science (SPSS), software version 23.0 were used to collect and analyze the data (frequencies and percentages).

### Quality assessment

The Van Tulder Scale [26] for reporting randomized controlled trials and the Downs and Black checklist [27] for assessing the methodological quality of non-randomized research has been used to evaluate the quality of included studies by two reviewer authors separately. We graded each non-randomized trial as ‘excellent, good, fair, or bad,‘ while we categorized each randomized study as low quality if it had 5 points or high quality if it had 5 points based on Van Tulder’s modified classification.

## Study results

### Study selection

The initial search identified 431,753 studies and key parameters were chosen to contribute to the selection of studies: (keywords, type of study, date, full text, language, and title) Table 1. Subsequently, the exclusion of title, abstract and duplicates assessment, 155 articles were evaluated, which yield 129 studies for full-text reading articles and to be the excluded studies 26 because of study design, not located in one of the Middle East countries, no relevant aim and not meet the other inclusion criteria. At the conclusion of the selection process, 129 articles matched the requirements for inclusion Fig. 1.

**Table 1:** Search results from different electronic databases

Name of Database	Parameters	Number of studies
PubMed	Keywords = 36,403 Types of study = 1117 Date = 841 Full text = 824 Language = 824 Title = 40	40,049
Scopus	Keywords = 171,334 Types of study = 31,415 Date = 23,720 Full text = 18,835 Language = 18,390 Title = 74	263,768
Cochrane Library	Keywords = 106 Types of study = 104 Date = 104 Full text= ------ Language= ----- Title = 44	358
Springer Link	Keywords = 43,030 Types of study = 28,957 Date = 16,779 Full text = 16,779 Language = 16,712 Title = 72	122,329
Clinical Trial	Keywords = 2 Types of study = 2 Date = 2 Full text= ------ Language= ----- Title = 2	8
Web of Science	Keywords = 1576 Types of study = 1447 Date = 1095 Full text= ------ Language = 1084 Title = 39	5241

### Study characteristics and journal of publication

The study characteristics of the included studies have listed in **Supplementary file.** The selected studies were published between 2013 and 2020 and the studies were conducted in Jordan, with the largest number of published studies (25.6%, n = 33) followed by Saudi Arabia (16.3%, n = 21), Turkey (11.6%, n = 15), Iran (10.9%, n = 14) then Egypt (9.3%, n = 12), Qatar (6.2%, n = 8),Cyprus (5.4%, n = 7), Iraq (4.7%, n = 6) and Kuwait (3.1%, n = 4) While Lebanon and Palestine (2.3%, n = 3) next Israel and Oman (1.6%,n = 2, and 0.8%, n = 1, respectively). Regarding the year of publication, we found that the majority of studies were published in 2020 by 25.6% followed by2018 = 17.1%, and 14% for the studies published in 2017 and 2016. While 7% for the ones published in 2013 and 6.2% for 2015, then 3.9% for the studies published in 2014 as shown in Table 2.

**Table 2:** The frequency distribution and the total percentage of included studies and their year of publication

		Frequency	Percent
Country	Jordan	33	25.6%
	Iran	14	10.9%
	Egypt	12	9.3%
	Iraq	6	4.7%
	Cyprus	7	5.4%
	Qatar	8	6.2%
	Saudi Arabia	21	16.3%
	Turkey	15	11.6%
	Israel	2	1.6%
	Oman	1	0.8%
	Lebanon	3	2.3%
	Kuwait	4	3.1%
	Palestine	3	2.3%
	**Total**	**129**	**100.0%**
		Frequency	Percent
Year	2013	9	7%
	2014	5	3.9%
	2015	8	6.2%
	2016	18	14%
	2017	18	14%
	2018	22	17.1%
	2019	16	12.4%
	2020	33	25.6%
	**Total**	**129**	**100.0%**

According to the study design of the selected studies, the majority of them were non- randomized controlled trials (66.7%, n = 86) including four types of prospective, pre-post interventional, retrospective, and cross-sectional studies, and (33.3%, n = 43) studies were identified as Randomized Table 3.

**Table 3:** The type of study design applied in the Included studies (n = 129)

		Frequency	Percent
Design	Randomized Controlled Studies	43	33.3%
	Non-Randomized studies	86	67.4%
	Total	129	100%

On the other hand, the most common type of journals used to publish full reports of data of included studies was an International Journal of Clinical Pharmacy and subsequently followed by the Journal of Oncology Pharmacy Practice, Jordan Journal of Pharmaceutical Science, and Journal of Evaluation in Clinical Practice (14%, 4.7%) Table 4. Whereas 3.9% from the included studies were published in Saudi Pharmaceutical Journal and 68.2% from the involved studies were published in various types of journals.

**Table 4:** The Type of Journals Used to Publish the Articles

		Frequency	Percent
Nameof Journal	International Journal of Clinical Pharmacy	18	14%
	Journal of Oncology Pharmacy Practice	6	4.7%
	Jordan Journal of Pharmaceutical Science	6	4.7%
	Journal of Evaluation in Clinical Practice	6	4.7%
	Saudi Pharmaceutical Journal	5	3.9%
	Other Type of Journals	88	68.2%
	**Total**	**129**	**100%**

### Nature of pharmaceutical care services provided

The main outcomes measured were as follows: Identification of DRPs and disease progression (49.7%, n = 95), increase in patient education, medication adherence, and improvement in QoL (41.4%, n = 79), and 7 studies with 3.7% for mortality and reduction in hospitalization. While four studies (2.1%) focused only on the cost- effectiveness of pharmaceutical care services and 6 studies (3.1%) on determining the barriers encountered in the use of pharmaceutical care services in the region of the Middle East Table 5. On the other hand, most of the units in which the pharmaceutical intervention was applied are respectively as follows: Community pharmacies by 17.9%, Cardiac clinic = 12.9%, Oncology = 8.6% and Out-patients clinic, DM clinic and Internal = 5.7%, 5.7%, and 5%, respectively. 3.6% for Haematology and Pediatrics clinics. On the other hand, 2.9% were conducted in ICU, Psychic clinic, Geriatrics, ED, Multiple centers, and Infectious wards, while 1.4% in Nephrology clinic, not specified, Ambulatory pharmacy Syrian refugees’ clinic, Gynecology, and Neurology. Correspondingly, 0.7% had focused on the intervention of pharmacists/ clinical pharmacists in DIC, Urology, HIV clinic, chronic diseases clinic, Kidney transplantation clinic, and Anticoagulation clinic Table 6.

**Table 5:** The outcomes achieved through the implementation of pharmaceutical care services in different Middle East countries

		Frequency	Percent
Outcomes	IdentificationofDRPsand	95	49.7%
	Disease Progression		
	IncreasePatientEducation,	79	41.4%
	Adherence,andImprove		
	QoL/HRQoL		
	ReduceMortalityand	7	3.7%
	Hospitalization Rates		
	Cost-effectiveness of PCs	4	2.1%
	Barriers hinder PCs	6	3.1%
	**Total**	**191**	**100.0%**

**Table 6:** List of medical facilities that had received the services of the pharmacy practice

		Frequency	Percent
Units	Drug Information Center (DIC)	1	0.7%
	Nephrology	2	1.4%
	Intensive Care Unit (ICU)	4	2.9%
	Cardiac Unit (Rehabilitation)	18	12.9%
	Outpatient clinics	8	5.7%
	Psychiatric unit	4	2.9%
	Geriatrics unit	4	2.9%
	Urology clinics	1	0.7%
	Diabetes clinics	8	5.7%
	Internal Ward	7	5%
	Hemodialysis unit	5	3.6%
	Pediatrics unit	5	3.6%
	Oncology Unit	12	8.6%
	Community Pharmacy	25	17.9%
	EmergencyDepartmentand Hospitalized	4	2.9%
	Multiple centers	4	2.9%
	Not specified	2	1.4%
	Ambulatory Pharmacy	2	1.4%
	Syrian Refugees’ clinic	2	1.4%
	HIV center	1	0.7%
	Government and private hospitals	10	7.1%
	Gynecology	2	1.4%
	Infectious ward	4	2.9%
	Chronic disease ward	1	0.7%
	Kidney transplantation clinic	1	0.7%
	Anticoagulation clinic	1	0.7%
	Neurology	2	1.4%
**Total**		**122**	**100.0%**

### Pharmaceutical services implementers

**Fig. 2:**
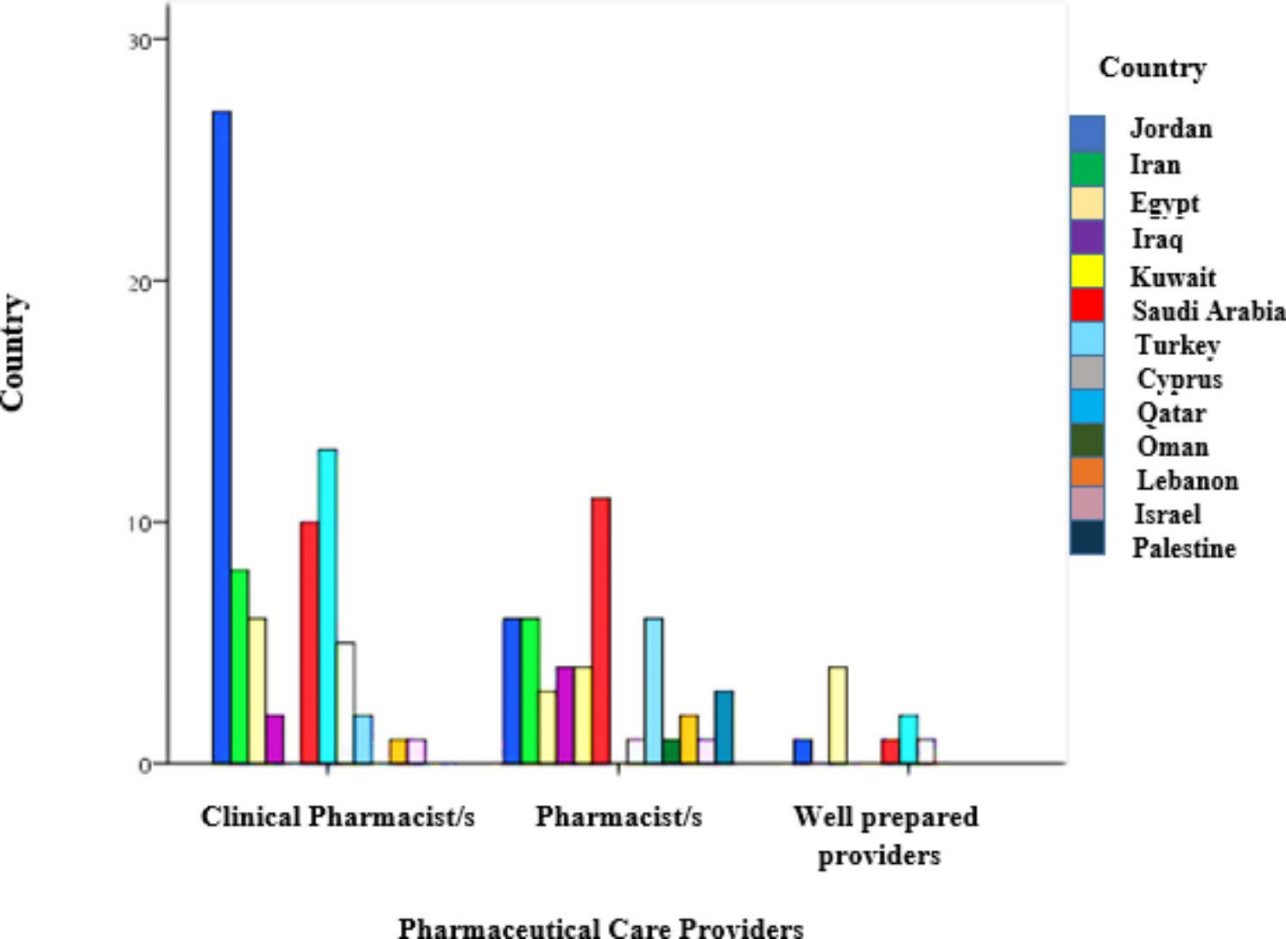
A comparison of people in charge of providing pharmaceutical care services in several Middle Eastern countries

In terms of the implementation of the pharmaceutical interventions, we found that most of these interventions were by clinical pharmacists by 57%, 34.8% by pharmacists, and followed by 8.1% for well-prepared health practitioners. Where we found that one of the most countries to have a pharmaceutical intervention or provide services through the clinical pharmacist is Jordan by 36.6%, then Turkey by 17.3% and Saudi Arabia by 13.3%, followed by Iran, Egypt and Cyprus by 10.7%, 8%, and 6.7%, respectively. Lastly, 2.7% for Iraq and Qatar. While we observed that most countries relied on pharmacists to provide pharmaceutical services was Saudi Arabia by 22.9%, followed by Jordan, Iran, and Qatar by 12.5%, then 8.3% for Iraq and Kuwait. As well as, by 6.3% for Palestine and 4.2% in Lebanon. While 2.1% for Israel, Oman, and Cyprus, sequentially. Besides, in Egypt by 44.4%was depending on well health practitioners, followed by 11.1% for Jordan, Cyprus, and Saudi Arabia, respectively Fig. 2.

### Study quality

The majority of the included studies were rated as high quality because they were randomized, and all of the high-quality research included a quantitative study design. While the majority of non-randomized studies are thought to be of medium quality Table 7.

**Table 7:** Comparison of the types of study designs and the quality rate of included studies

			Quality				Total
			High	low	Good	fair	
Design	Randomized Controlled Studies	Number	37	6	0	0	43
		%within design	100%	100%	0.0%	0.0%	33.3%
	Non-Randomized studies	Number	0	0	21	65	86
		%within design	0.0%	0.0%	100%	100%	66.7%
**Total**		**Number**	**37**	**6**	**21**	**65**	**129**
		**%within design**	**100%**	**100%**	**100%**	**100%**	**100.0%**

### The implication of included studies in relation to pharmaceutical services

Through the data analysis, we found that most of our selected studies confirmed the vital role of clinical pharmacists or pharmacists by 58.5% in different ways and implementing different services. Moreover, 21.5% of doctors confirm cooperation with clinical pharmacists or pharmacists to provide various pharmaceutical care services.

**Fig. 3:**
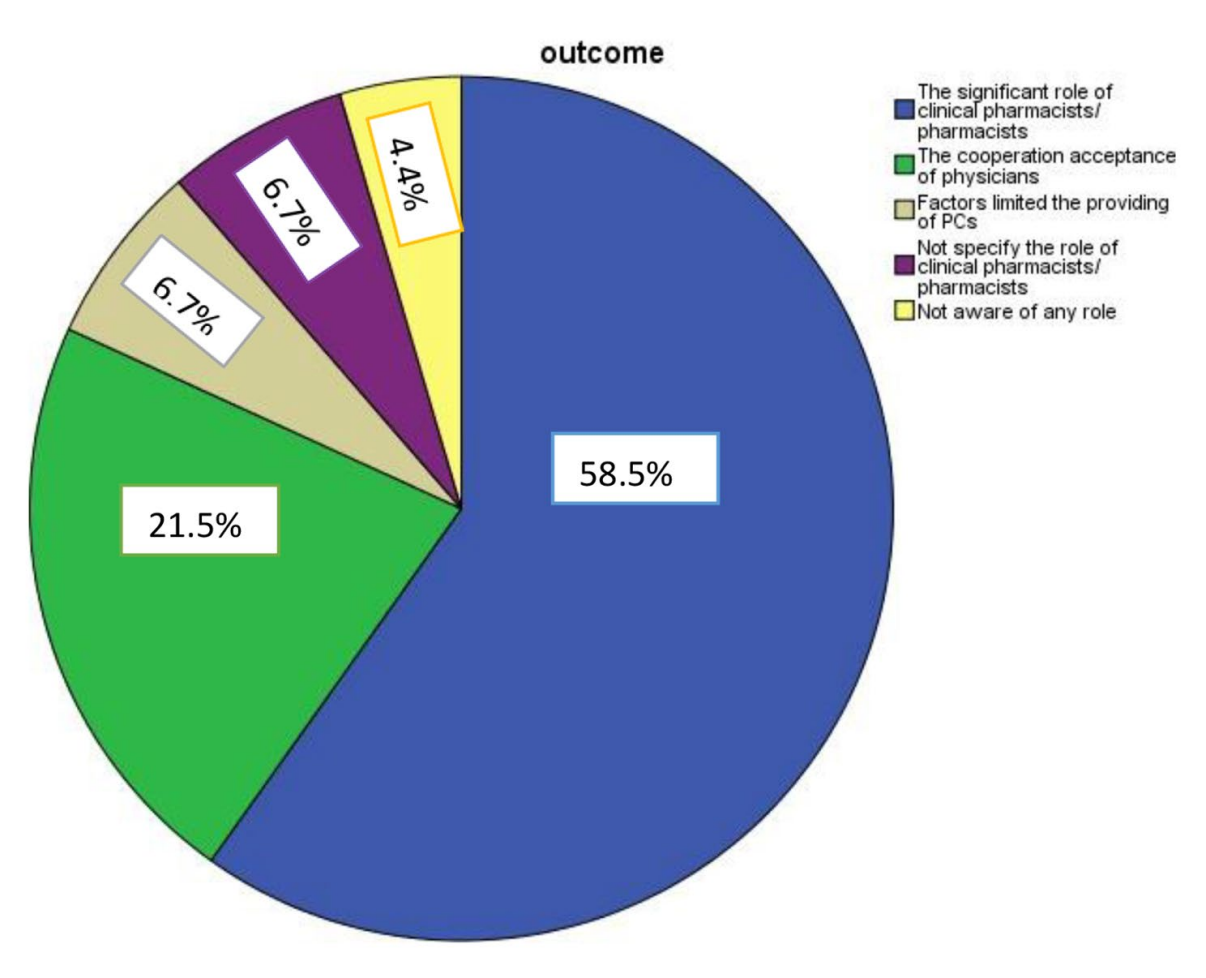
The feedback of studies regarding the implementation of pharmaceutical care services

On the other hand, 6.7% of some selected studies, we discovered these studies mentioned the factors that may limit the implementation of PC services. Nevertheless, 6.7% of some studies have not given any consideration to the role of clinical pharmacists or pharmacists because they were exploring the role of the devices applied, while 4.4% demonstrated that there is no role for the clinical pharmacists or pharmacists. Figure 3.

### The impact of pharmaceutical care services provided

#### Identification of DRPs and diseases progression

Many pharmaceutical care services that have been covered by clinical pharmacists or pharmacists have been identified in various aspects. Getting back to the key aim of this review, thirty-three of the studies conducted in different Middle East Countries including Jordan (22.2%, n = 8), Cyprus (8.3%, n = 3), Israel (2.8%, n = 1), Qatar (8.3%, n = 2), Saudi Arabia (8.3%, n = 3), Oman (2.8%, n = 1.3), Turkey (25%, n = 7), Egypt (11.1%, n = 4), Iran (5.6%, n = 2) and Lebanon (5.6%, n = 2) showed how the clinical pharmacists/ pharmacists play a fundamental role to assess the patient’s medications treatment from different aspects and different unites in the hospital which is facilitating the treatment control and enhancing therapy outcomes. Also, further studies [28–30] showed a great acceptance of physicians for clinical pharmacists’ recommendations regarding their intervention in identifying drug-related problems. The intervention of clinical pharmacists in reducing and identifying drug-related problems resulted in a significant impact in detecting drug problems and preventing harm [31–35]. While one study conducted in Turkey highlighted that despite the non- properly implementation of clinical pharmacy services, clinical pharmacists have a high rate of acceptance in recommending DRPs in a wide variety [36].

In a community, pharmacists are the most accessible health care provider. As a result, they are in a position to detect chronic diseases early on and identify unhealthy behaviors. When appropriate, they can assist patient’s lower risk factors by providing prevention counseling, such as weight and food control, exercise, and quitting smoking [1]. As a result, we investigated

21.2% of research that examined the role of a clinical pharmacist or a pharmacist in managing, regulating, and enhancing a patient’s health. Clinical pharmacists/ pharmacists have a considerable impact on monitoring and maintaining clinical parameters of diabetic patients, according to 8.3% of studies in the field [37–44]. Respectively, we found that 6.8% of the studies focused on the positive role of pharmacists/ clinical pharmacists in different diseases such as metabolic syndrome, Poly Cystic Ovary Syndrome (PCOS), iron deficiency anemia and others [45–53].

Furthermore, 2.3% of the studies related to the managing of Cardio Vascular Diseases [54–56](CVDs), while 2.3% of the studies focused on the intervention on patients with Chronic Kidney Diseases (CKDs) [10, 57, 58] and 1.5% of the studies described the implementation of PCs in patients with respiratory diseases such as asthma [59, 60]. All of them substantiated that pharmacists/ clinical pharmacists are an essential part of the health care team to provide early detection of chronic diseases, managing associated complications, and how the physicians strongly recommended the importance of integrating the pharmaceutical care services.

According to PCNE, each problem can have many causes, with each problem having a maximum of three causes [61]. The classification will be used in research into the nature, prevalence, and incidence of DRPs, as well as in experimental studies of Pharmaceutical Care outcomes as a process indicator. It’s also designed to make it easier for healthcare workers to keep track of DRP data during the pharmaceutical treatment process. Lack of knowledge about medications, lack of adherence to medications, suboptimal efficacy of medications prescribed, untreated conditions, selection of doses, inadequate training, lack of space for counseling, and the need for additional monitoring were all identified as DRPs that could affect the sustainability of PCs and the progression of chronic diseases in some of the included studies [10, 58, 62].

#### Patient education, medication adherence, and improvement in QoL/ HRQoL

Regarding the role of pharmacists/ clinical pharmacists in counselling, educating, enhancing medications used, and improving (QOL) of the patients, we found that 41.4% of the studies showed their great impact, well-defined education, care that is helping to improve the outcomes of the patients and increase their desire to counsel, complete their medications appropriately and the positive impact of counselling not only on medication use but to develop a variety of strategies to improve compliance too. Where the percentage of studies that focused on increasing adherence to the use of the drug was 17.3%, 21.1% were focused mainly on the role of pharmacists/ clinical pharmacists in educating and counselling, and 20.3% on their effective role in improving the quality of life of the patients. Besides, some studies demonstrated that comprehensive educational intervention might improve the knowledge, attitudes, and perceptions among pharmacists and encourage them to incorporate and participate in this into their everyday clinical practice.

#### Mortality and hospitalization rates

From other aspects, we discovered that 3.7% of the studies confirmed the significant impact of pharmaceutical care services and the great reductions in the rate of hospitalization. In addition, following the pharmacist’s guidance and recommendations resulted in a considerable drop in mortality. A study conducted by Muath Fahmi Najjar, suggested that the intervention of clinical pharmacists would raise the significance value of their intervention to improve the prescription patterns among hospitalized patients [63]. Moreover, another study linked the intervention of a clinical pharmacist to the reduction of hospitalization, thus reducing the high cost that the patient may incur as a result of re-admission to the hospital [64].

#### Cost-effectiveness of pharmaceutical care

11.2% of the studies focused on the role of clinical pharmacists/ pharmacists in detecting irrational drug utilization, reducing medications cost either for hospitalized or non-hospitalized patients. This research found that clinical pharmacists have a considerable impact on drug therapy costs, as well as reducing inappropriate prescription use at admission and improving medication review services. Clinical pharmacist interventions did not significantly reduce patients’ direct drug costs, according to a study by Hossein Khalili, [65]. Clinical pharmacist intervention has a considerable impact on the cost of pharmacological therapy and patient outcomes, according to a study conducted by Huda, [66]. The findings back up the value of pharmacological therapy for all hospitalized CVD patients. A retrospective study had done by Elif Ertuna, despite clinical pharmacy services still not properly implemented in Turkey; there’s a high acceptance rate of pharmacist recommendations in interdisciplinary geriatric assessment teams [36]. Based on the results conducted in a reference center in Iran, the implementation of guidelines by the pharmaceutical supply unit significantly reduced the consumption of albumin and IV pantoprazole and decreased their direct costs [67].

As a result of the continually high cost of pharmaceuticals, Pharmacoeconomics evaluation studies are becoming increasingly important [68]. These studies allow for the identification, measurement, and comparison of the expenses of various pharmacotherapies or services, as well as the assessment of their impact on health budgets and patient health.

### Barriers hinder pharmaceutical care services

One of the goals that ensure continued Pharmaceutical Services is to secure patient satisfaction for services provided by either the pharmacist or the pharmacist’s clinical. Two studies [69, 70] showed that all the patients considered counseling as an important service and were satisfied with it and how pharmacist with skills in pharmaceutical care and counseling could be useful in promoting the service and making it profitable for the pharmacy business as shown too in this study that all the participants had a positive perception of the pharmaceutical care services.

Moreover, pharmacists/ clinical pharmacists may face some barriers and challenges to provide pharmacy practice, so different studies [71–73] demonstrated that time constraint’ was the primary barrier discouraging pharmacists from practicing such service, inconvenient access to patient medical information (78%) and lack of staff and time (77 and 74% respectively) and research experience for the pharmacists who have no prior experience to do research.

As a result, two studies concluded that collaboration among many stakeholders in the profession is critical to supporting pharmacists’ attempts to change the scope of pharmacy practice in order to improve patient care services. Furthermore, from undergraduate studies to residency training, pharmacy practice implementation is influenced by the entire educational process [73, 74].

## Discussion

The current research is the first to examine the impact of PCs on Middle Eastern low- and middle-income countries.

Only 129 articles were included in this scoping review. However, all included studies have been published from 2013 to 2020. The massive majority of studies were from Jordan, with the largest number of published studies (25.6%, n = 33), followed by Saudi Arabia (16.3%, n = 21), Turkey (11.6%, n = 15), Iran (10.9%, n = 14) then Egypt (9.3%, n = 12), Qatar (6.2%, n = 8), Cyprus (5.4%, n = 7), Iraq (4.7%, n = 6) and Kuwait (3.1%, n = 4) While Lebanon and Palestine (2.3%, n = 3), next Israel and Oman (1.6%, n = 2 and 0.8%, n = 1, respectively). Based on the World Bank Classification of countries by income level 2019–2020 (World Bank Data 2020), we found thirteen Middle East Countries. Six of them were identified as Middle-Income countries including Jordan, Iran, Egypt, Iraq, and Turkey, six countries considered as High income representing in Qatar, Cyprus, Saudi Arabia, Israel, and Oman, and one country Palestine was considered a Low-income country.

Most of the studies included in this review reported the effective role of pharmacist/ clinical pharmacists in improving HRQoL/ QoL. This finding is also compatible with results from previous studies, which showed significant value and the positive impact of pharmacists on the QoL among older adult patients in rural areas[75]. Moreover, a study conducted in 2014 recited a statistical improvement in the HRQoL after pharmaceutical care intervention[76]. A review supports this systematic review was carried on women with breast cancer (BC) in the Middle East countries [77]. A cross-sectional study conducted by Jordanian pharmacists to determine the predictors, levels, and prevalence of anxiety and stress, as well as the relationship between these factors and quality of life in recently displaced Iraqis, discovered that pharmacists play an important role in reducing anxiety and stress among refugees. Reducing and managing anxiety and stress may help refugees around the world improve their quality of life [78]. While we found that some studies have traded different services that can be provided by pharmacists or clinical pharmacists. Majdoleen AL Alawneh, showed that the medication review service provided by professional pharmacists can enhance DRPs and anxiety ratings, according to this study [79].

Similar results were found in different studies. Pharmacists are capable of assisting patients in improving their health by lowering drug-related side effects and increasing medication adherence, as well as reducing physician visits, and hospital admissions, and changing the primary care delivery system as a whole [80]. A systematic review correlated to pharmacist- led DM self-management Education (DSME) studies also indicated a significant improvement in medication adherence, quality of life, and diabetes knowledge after DSME [81].

In a systematic review of non-dispensing pharmaceutical services in low- and middle-income countries, Pande et al. (2013) found that “pharmaceutical care” was the most prevalent language used in the studies, and the majority of the interventions involved simple patient education [82].

Although the main services were implemented either more specific, such as improving HRQoL/QoL, managing and controlling diseases, increasing medication adherence, resolving DRPs, or TRPs. The effectiveness of CPS delivered in primary care clinics was evaluated in a systematic review, and the majority of the complex and comprehensive interventions, such as physical assessment, monitoring, prescribing, and face-to-face communication with physicians, were conducted in high-income countries [83]. Undoubtedly, the majority of congestive heart disease (CHD) seems to be closely related to abnormal BP, diabetes, and dyslipidemia [84], in which several studies have been shown the effective role of pharmacists in reducing morbidity and mortality associated with CHD, as well as numerous articles have been identified that cooperation, especially cooperation between physicians and pharmacists, can effectively reduce the incidence of drug-related problems and improve outcomes very favorably in some clinical situations [85–87].

The need for knowledgeable, proficient, and experienced healthcare practitioners has grown year after year and the role of a clinical pharmacist has expanded to encompass all phases of patient care as part of the healthcare team. A study looking at physicians, nurse practitioners, and physician assistants estimated a shortage of qualified oncology and Hematology practitioners by 2020 [88]. Because of their special knowledge and extensive training, oncology pharmacists are perfectly positioned to deliver high-quality care to cancer patients and offset some of the shortage of practitioners [89]. Another study conducted in the Hematological and Has Unit revealed that the inclusion of a clinical pharmacist in the hemodialysis unit resulted in the detection and treatment of several DRPs. The majority of the interventions were important, and they may have resulted in improved therapeutic outcomes [90]. Additionally, other studies conducted in diabetes clinics have documented the diabetes management of patients whose drug therapy was managed specifically by clinical pharmacists under physician supervision. The studies showed consistent, favorable results on glycemic control in the university-affiliated out-patient clinic, Veterans Affairs medical centers, and managed care settings [91–94].

The existence, accessibility, and inference of hospital pharmacists need to be improved, and physicians should be more aware of what they can offer, as shown in our scoping review. Qualitative semi-structured interviews revealed that the presence, visibility, and implication of hospital pharmacists need to be improved, and physicians should be more aware of what they can offer, according to qualitative semi-structured interviews. As a result, trust is one of the most important factors for improving physician–pharmacist collaboration, as numerous studies discovered [95–97]. Trust is critical for physician–pharmacist collaboration and has been linked to greater commitment and dialogue.

According to our analysis, we attempted to identify elements that could either encourage or hinder the introduction of pharmaceutical care services in Middle Eastern countries. Hence, we found only a few studies that reported some factors, including a limited number of clinical pharmacists, lack of cooperation between pharmacists/ clinical pharmacists and health care professionals, low variable socioeconomic status, and lack of expert and trained pharmacists. A study reported that the identification of barriers and facilitators of implementation is one among the foremost important and feasible strategies to implement change. Therefore, the identification of influencing factors employing a framework, like APOTECA, could guide the development of a multifaceted, multilevel tailored plan, using implementation strategy tools, to a successful implementation of CPS [98].

The cost-effectiveness of pharmaceutical care interventions aims either to reduce medication errors or to reduce the cost of medication-related morbidity and mortality. As a result, there is a great opportunity for pharmacists to have a significant impact on reducing healthcare costs because they have the expertise to identify, correct, and prevent medication errors and medication-related problems. For this reason, two studies have examined the cost-effectiveness of clinical pharmacists providing services in a general practice (GP) [99, 100]. Pharmacist intervention dominated, decreased costs, and improved health outcomes, according to the Canadian Cancer Society in Canada [101]. In a study conducted in the United Kingdom, pharmacist intervention was found to be more cost-effective than conventional care [102].

Because not all Middle Eastern countries were covered in the scoping review, there are certain limitations on how far the influence of pharmaceutical care services may be generalized. Additionally, we were unable to examine pharmaceutical care services in various Middle Eastern nations due to a lack of research and other nations’ rejection of interventions. While some research emphasized the value of cost-effectiveness, the majority of studies conducted in the Middle East did not, which may be one of the challenges to accessing pharmaceutical therapy.

To sum up, this scoping review showed that Clinical pharmacist’s/ pharmacists’ interventions in different general practices have a significant role in improving healthcare outcomes, including QoL, medication adherence, mortality rate, hospital readmission, and hospitalization, reviewing patients’ medication discrepancies, and DRPs. These data can be used to design a strategy that identifies the factors that assure the continuation of pharmaceutical services as well as the issues that hinder this continuity. Furthermore, it may be possible to provide these services in nations where Pharmaceutical Care Services are not unified, thus consider including them.

## Electronic supplementary material

Below is the link to the electronic supplementary material.

Supplementary Material 1

## Data Availability

The datasets used and/or analysed during the current study are available from the corresponding author on reasonable request.
