# Supplementary material for: Evaluation of pharmaceutical care services in the Middle East Countries: a review of studies of 2013–2020
Source: BMC Public Health. 2023 Jul 17;23:1364. doi: 10.1186/s12889-023-16199-1 (PMC10351150; doi:10.1186/s12889-023-16199-1)
Supplement: Supplementary file 1 — Supplementary Material 1 [file 12889_2023_16199_MOESM1_ESM.pdf]

Supplementary File : *Characterizations of the studies (n= 129)*

Authors/ Year	Location	Design	Hospital characterization	Intervention by	Unit	Data collection	Data analysis	PCs Implemented	Quality rating	Name of Journal	Impact Factor
Attalla Saleh Abu-Oliem et al. (2013)	Jordan	Randomized control trial	This ICU has 37 beds. An average of 35 patients was admitted every week from internal medicine, surgery, and traumatic patients (for example, road traffic accidents).	Clinical pharmacist	ICU	-The database was collected from patients' medical files.<br>-Interview with patients.<br>- Facilitated discussion with physicians.	Thematic analysis	Resolving and preventing DRPs.	High	Jordan Journal of Pharmaceutical Science	0.35
Linda M Tahaineh et al. (2018)	Jordan	A prospective parallel randomized trial.	Outpatients clinics	Clinical pharmacist	Outpatients clinics	Data collected through patients' interviews and verbal recommendations to physician's concerning the intervention group management plan.	Thematic analysis	Managing iron deficiency anemia patients.	High	International Journal of Pharmacy Practice	0.391
Yasmeen AbuNaba'a et al. (2019)	Jordan	A parallel randomized trial.	Not determined	Clinical pharmacist	Psychiatric clinic	Data collected through patients' interviews and recommendations discussed with physicians and quaternary was made.	Descriptive analysis.	Counseling and education regarding treatment, adherence, and self-care activities	High	Journal of Evaluation in Clinical Practice	1.681
Chung-Ying Lin et al. (2017)	Iran	Randomized control trial	(Five academic centers in Tehran, two in Qazvin and Ahvaz each, one in Semnan,	Well prepared practitioners.	5 multiple centers	Facilitated discussion groups and focus group	Multilevel linear mixed models	Medication adherence, improving QoL,	High	Drugs and Aging Journal	2.381

			Zanjan, and Tabriz each).					and reducing the mortality rate.			
Salma M Bahnasawy et al. (2017)	Egypt	A prospective randomized study.	Not determined	Clinical pharmacist	Pediatric clinic	By using the Generic Core Scale Questionnaire and an interview with patients.	Statistical analysis	Increasing awareness regards iron overload and enhancing the patient's outcome.	High	Basic and Clinical Pharmacology and Toxicology	3.176
Rajaa A Al-Qudah et al. (2020)	Jordan	A prospective randomized study.	Outpatient clinic setting of the Jordan University Hospital in Amman, Jordan, the main health care provider in Jordan, with 600 beds and serving over 500 000 outpatients annually.	Clinical pharmacist	Outpatient clinic	Patient interview method via a questionnaire	Statistical analysis	Resolving TRP among patients with chronic diseases.	High	Journal of Evaluation in Clinical Practice	1.681
Zahra Jahangard-Rafsanjani et al. (2015)	Iran	Randomized control study	Not determined.	Community pharmacist	Endocrine clinic	5 follow-up visits and 5 phone calls) and recommended physician visits when necessary.	Statistical analysis	Developing and delivering a diabetes support program for patients	High	The Science of Diabetes self-management and Care Journal	2.087
EylemIlktac Korcegez et al. (2017)	Cyprus	Randomized control study.	The study was conducted with 152 patients at a 186-bed public hospital's outpatient diabetes clinic in Gazimagusa, Northern Cyprus.	Clinical pharmacist	Outpatient diabetic clinic	Data were collected using questionnaires, medical reports, and the hospital's record system.	Statistical analysis	Achieving treatment goals in patients with DM, improving pts. outcomes, and medication adherence.	High	Journal of Managed Care and Specialty Pharmacy	2.713
Maguy Saffouh El Hajj et al. (2017)	Qatar	Prospective randomized control study.	Not determined.	Pharmacist	Ambulatory pharmacies	Face to face structure interview.	Statistical analysis	Implanting and delivering the smoking program, reducing the rate of death, and preventing respiratory disease.	High	BMC Public Health	2.948

Amir Sarayani et al. (2018)	Iran	Parallel randomized control study.	Tehran University of Medical Sciences (conference hall and the drug information call center). This pharmacy is in the midtown area of Tehran, the capital city of Iran, and is the main pharmacy among 7 educational pharmacies affiliated with the College.	Pharmacist	DIC	Phone calls and discussion with physicians	Statistical analysis	Improving patient care activity, medication adherence, and DM parameters.	High	International Journal of Clinical Pharmacy	1.692
Mera Ababneh et al. (2019)	Jordan	Prospective randomized control study.	Not determined	Clinical pharmacist	Urology clinic	Verbal communication with patients, questionnaires, and some discussion was with physicians	Statistical analysis	Increasing medication adherence, education of patients, and improving lifestyle.	Low	International Journal of Clinical Pharmacy	1.616
Maryam Mehrpooya et al. (2018)	Iran	Randomized control study	Not determined.	Pharmacists	Cardiac unit	Face to face and Morisky questionnaire	Statistical analysis	Increasing adherence to medications and educating the patients about their medications and disease.	Low	Journal of Applied Pharmaceutical Science	0.62
Amani Zidan et al. (2016)	Qatar	Prospective randomized control study	The center is equipped with a 20-bed coronary care unit, a 12-bed cardiothoracic intensive care unit, a 24-bed surgical high dependency unit, and 60 ward beds with continuous telemetry monitoring rooms.	Clinical pharmacist	Cardiology unit	By reviewing the HH’s medical records according to the study's inclusion and exclusion criteria.	Statistical analysis	Reviewing and reconciling patient's discharge medications, Documenting and resolving any identified discrepancies and DRP, and Providing the usual general	High	BMJ Open	2.496

								discharge counseling.			
Haitham Saeed et al. (2020)	Egypt	Randomized control study.	Not determined.	Well prepared practitioners	3 different clinics	A smartphone application was recorded with verbal communication.	Statistical analysis	Improving and educating patients about their use of the selected device.	Low	Journal of Respiratory Care	2.012
Maryam Mousavi et al. (2013)	Iran	Randomized control study	Not determined.	Clinical pharmacist	Transplantation ward.	Daily services were applied to patients according to the patient's health status	Statistical analysis	Providing well PN, maintain the natural status of patients, and improving clinical outcomes.	High	Journal of Support Care in Cancer	2.698
Amna Al-Hashar et al. (2018)	Oman	Randomized control study.	A tertiary care academic hospital in Oman with a bed capacity of 500.	Pharmacist	Internal medical ward	Electronic health records and interviews.	Statistical analysis	Reducing medication errors and reconciliation on admission and discharge.	High	International Journal of Clinical Pharmacy	1.692
Ruba A Wishah et al. (2015)	Jordan	Randomized control study.	The diabetes clinic at JUH provides usual care services to more than 90 patients daily with regular follow-up clinic visits every 1–3 months, depending on the glycemic control for each patient.	Clinical pharmacist	Diabetes clinic	By using the structured interview guide, medical charts, and hospital computers.	Descriptive analysis	Controlling diabetic type 2 patients and evaluating patients' adherence to medication.	High	Diabetes and Metabolic Syndrome: Clinical Research and Review	0.672
K Aljumah et al. (2015)	Saudi Arabia	Randomized control study	This city has a total population of more than 5,000,000 and one psychiatric hospital (Al-Amal Hospital; a total of 500 beds) is the main provider of psychiatric	Pharmacist	Psychiatric clinic	Applying intervention by a pharmacist and using a questionnaire.	Descriptive and comparative analysis	Improving adherence to medication, patient's outcome, and improving patient's knowledge.	High	BMC Psychiatry	1.369

			care for the entire population.								
Lana K. Salameh et al. (2019)	Jordan	Randomized control study	At the Jordan University Hospital (JUH), a 550 beds' tertiary teaching hospital located in Amman, the capital of Jordan.	Pharmacist	Internal medical ward	Data collected from the patients' medical records, followed by interviewing the patient	Statistical analysis	Reducing medication discrepancies and improving patients' outcomes.	High	Journal of Pharmaceutical Health Services Research	0.287
Radwa El Borolossy et al. (2014)	Egypt	Prospective randomized control study	Not determined	Clinical pharmacist	Hemodialysis clinic	Applying intervention by a pharmacist and using a questionnaire.	Statistical analysis	Reducing the risk of developing DTRPs	High	Journal of the International Pediatrics Nephrology Association	2.676
Zina Tahsin Ali et al. (2019)	Iraq	Randomized control study	Not determined	Pharmacist	Endocrine clinic	Face to face interview, telephone call, and questionnaire	Not reported	Improving, reviewing a patient's health parameters and enhancing medication adherence	High	Annual Tropical Medicine and Public Health	0.131
Dvora Frankenthal et al. (2014)	Israel	Parallel randomized control study	The facility has 384 beds and is staffed by five physicians and one chief physician who is the medical director of the facility.	Pharmacist	Geriatric unit	Reviewing patients' medications and using specific scales.	Statistical analysis	Detecting inappropriate prescription, right treatment, and medications, enhancing clinical outcome and improving QoL	High	Journal of American Geriatric Society	4.388
Eman Ahmed Casper et al. (2019)	Egypt	Prospective randomized control study	Not determined	Clinical pharmacist	Cardiac rehabilitation unit	Data collected via both patient record and patient interview	Statistical analysis	Reducing DRPs, enhancing drug therapy and clinical outcome	High	Basic and Clinical Pharmacology and Toxicology	2.651

Yassin, Harb Taha et al. (2020)	Iraq	Prospective randomized clinical trial	Not determined	Clinical pharmacist	Cardiac and internal wards	Motivational interview, medication review	Statistical analysis	Reducing re-hospitalization, discrepancies and improving HRQoL and adherence to medications	Low	Journal of Systematic Review Pharmacy	2.479
Bonyan Qudah et al. (2016)	Jordan	Randomized control study	Not determined	Clinical pharmacist	Hemodialysis unit	By automatic BP monitors that were equipped with the Fresenius® dialysis machines	Statistical analysis	Managing and controlling BP in hemodialysis patients	High	International Journal of Clinical Pharmacy	1.555
Taha O. Mahwi et al. (2013)	Iraq	Prospective, randomized control study	Not determined	Pharmacist	Diabetes clinic	Data of each patient were recorded in the patient data collection forms, questionnaire.	Statistical analysis and analytical analysis.	Controlling patients' parameters and reducing DTPs	Low	International Journal of Pharmaceutical Science and Research	0.27
Hend K. Eldeib et al. (2019)	Egypt	Prospective, randomized control study	Not determined	Pharmacist	National cancer institute	Using telephone follow up	Not reported	Enhancing medication adherence, the safety of oral chemotherapy, patient survival, and utilizing health outcomes.	High	Journal of Telemedicine and e-Health	2.385
Basima A Almomani et al. (2017)	Jordan	Randomized control study	Not determined	Clinical pharmacist	Pediatrics unit	The face-to-face interview was conducted and a questionnaire.	Statistical analysis	Controlling asthmatic health status and improving QoL	High	Journal of Patient Education and Counselling	2.607
Eman Ahmed Casper et al. (2016)	Egypt	Interventional randomized clinical trial	Not determined	Clinical pharmacist	Cardiac unit	Data collected via both patient record and patient interview	Statistical analysis	Enhancing patient outcome, educating patients, and increasing patient adherence to medications	Low	Basic and Clinical Pharmacology and Toxicology	2.651

Sewar S Salmany et al. (2018)	Jordan	Randomized control study	The study was conducted at a not-for-profit, 170-bed, teaching cancer center that treats about 3500 new cancer patients each year in both inpatient and outpatient settings.	Pharmacist	Oncology unit	Telephone follow up, medication chart, and Likert scale	Statistical analysis	Determining a patient's satisfaction after discharge.	High	Journal of Oncology Pharmacy Practice	1.603
Sumaya Abuloha et al. (2016)	Jordan	Prospective, randomized control study	The average number of patients visiting this clinic is about 70 patients/day, of these about 45 patients/day are diabetic, and most of them are T2DM.	Clinical pharmacist	Endocrine unit	Patients' medical records and questionnaire	Statistical analysis	Enhancing medication adherence, outcome, and educating patients about their health status and medications.	High	Jordan Journal of Pharmaceutical Science	0.35
Simin Dashti-Khavidaki et al. (2013)	Iran	Randomized control study	Not determined	Clinical pharmacist	Hemodialysis center	Patients' charts and prescriptions, interviews, and questionnaires.	Statistical analysis	Improving HRQoL, reducing the risk of hospitalization, and the rate of mortality.	High	International Journal of Clinical Pharmacy	1.25
Iman A Basheti et al. (2016)	Jordan	Prospective, randomized control study	At two community pharmacies in Amman, Jordan. On average, each community pharmacy serves 60–70 patients per day.	Clinical pharmacist	Community pharmacy	A validated pharmaceutical care tool was used, patient interviews, and by using the patient's medical records and questionnaire.	Statistical analysis	Assessing medication used by patients and managing TRPs.	High	International Journal of Clinical Pharmacy	1.555
Nour Faqee et al. (2020)	Jordan	Prospective study	KHCC is a 350-bed, internationally accredited cancer center in Amman, Jordan, which provides comprehensive care to adult and pediatric	Clinical pharmacist	Oncology unit	Routine review of patients' profiles and recorded CPIs, recording forms	Statistical analysis	Optimizing patient therapy and reducing DRPs.	Fair	Journal of Oncology Pharmacy Practice	1.85

			patients for all types of cancer in Jordan and the region.								
Naemeh Nikvarz et al. (2019)	Iran	Cross-sectional study	The study was conducted in a 20-bed nephrology ward of a tertiary teaching hospital affiliated to Kerman University of Medical Sciences (KUMS).	Clinical pharmacist	Nephrology ward	Survey	Statistical analysis	Improving VTE prophylaxis administration	Good	Europe Journal Hospital Pharmacy	0.892
Mesut Sancar et al. (2015)	Turkey	Pre-post intervention study	Not determined	Clinical pharmacist	Hospitalized and chest clinic	Using a list of accurate inhaler device use steps according to GOLD (the Global initiative for chronic Obstructive Lung Disease).	Statistical analysis	Increasing patient adherence to regular therapy and educating COPD patients on using inhalers in a hospital setting	Fair	Europe Journal Hospital Pharmacy	0.432
Tahrir Alnawayseh et al. (2020)	Jordan	Pre-post intervention study	Not determined	Clinical pharmacist	Internal medicine clinic – respiratory ward	A structured interview was conducted and Morisky Medication Adherence Scale	Statistical analysis	Educating on their use of inhaler device, enhancing medication adherence, and improving QoL	Good	Jordan Journal of Pharmaceutical Science	0.270
Ahmet S Boşnak et al. (2018)	Cyprus	Prospective study	The study was performed in the oncology department on in- and outpatients at Near East University Hospital, which is the biggest and leading medical facility in Nicosia, Northern Cyprus.	Clinical pharmacist	Oncology department	Appropriate interventions for each identified DRP were interviewed and discussed with the prescriber directly.	Statistical analysis and Graph Pad In Stat (version 3.00 for Windows 95, Graph Pad Software, San Diego California USA,	Identify the prevalence and nature of the DRPs encountered with the relevant factors and to understand the benefits to the treatment of the oncology pharmacists in this process.	Good	Journal of Oncology Pharmacy Practice	1.484

							www.graphpad.com).				
Birand Nevzat Et al. (2019)	Cyprus	Prospective study	Near East University Hospital is an institution affiliated with Near East University, Nicosia, and North Cyprus.	Clinical pharmacist	Oncology department	Patient education was provided face-to-face, and written information was tailored to the individual patient's chemotherapy protocol.<br><br>Beliefs about Medicines Questionnaire (BMQ) and The Morisky Green Levine Test (MGLT) were used	Statistical analysis	Evaluate patient medication adherence and beliefs and the potential impact of counseling by a pharmacist on cancer patient medication beliefs and adherence.	Good	Journal of Oncology Pharmacy Practice	1.85
Majdoleen AL Alawneh. Et al. (2020)	Jordan	Randomized Control Study	The study was conducted in three large Jordanian cities: Amman, Zarqa, and Mafraq, where The majority of the refugees from Syria residing in these cities.	Clinical Pharmacists	Syrian refugees	From the information collected, the pharmacist was able to identify the s for each participant. Besides, during home visits, two questionnaires were completed by the participants themselves, thus self-evaluating their QoL and anxiety scores	Statistical analysis	Evaluating Syrian refugee's QoL and anxiety scores.	High quality	Pharmacy Practice Journal	1.49
Yara R. Al Tall, Et al (2020)	Jordan	An exploratory qualitative study	The study was conducted at Forearms of Change Centre.<br><br>The Forearms of Change Center is located in Amman/Jordan and works on encouraging HIV/AIDS patients to	Trained researcher and qualified clinical Pharmacist	The center of HIV/ AIDS patients	A semi-structured questionnaire, so open and semi-structured questions were used in the interviews. Face-to-face interviews were conducted in private rooms to avoid any interruptions or distractions and to make participants comfortable.	-Recorded interviews were transcribed verbatim.<br><br>- Descriptive statistics	The barriers and facilitators of adherence to ART among HIV positive patients in Jordan and to assess their need for pharmaceutical care services.	Fair	The International Journal of Clinical Practice	2.444

			cohabit with their disease and give them positive support and help.							
Fadi Jarab, Et al. (2020)	Jordan	Retrospective study	Data were collected by referring to information technology departments at King Abdullah University Hospital.	Pharmacist	In patients who were clinically stable and received IV antibiotics for more than 24 hours	Data were collected retrospectively using electronic medical records and clinical notes, which included patient's file number, age, gender, height, weight, vital signs at admission, WBC count and serum creatinine at admission time, name of prescribed IV antibiotic and its dose regimen and duration, microbiology reports and sites of infection.	Descriptive statistics	Ensuring appropriate use of antibiotics in different hospital settings	Fair	The International Journal of Clinical Practice	2.444
Kinda T. Alkoudsi, Et al. (2019)	Jordan	Randomized control trial.	Community pharmacies (five pharmacies in Amman and five in Damascus).	Clinical pharmacists	Women diagnosed with PCOS	An intervention was done by Clinical pharmacists and then the Beck Anxiety inventory questionnaire was distributed.	Statistical analysis	The effect of a PCOS pharmaceutical care service on QOL for women diagnosed with PCOS living in Syria and Jordan	High	Journal of Education in Clinical Practice	1.681
Bushra Abdul Hadi, Et al (2015)	Jordan	Randomized control trial.	Specialty hospital in Jordan.<br><br>All the children were assessed for their ASA physical status.	-Surgeon<br>-Clinical Pharmacist	All the children were assessed for their ASA physical status.	- Provided the patients with simple information about the disease and drug therapy pre-, intra- and post-operatively.<br>- All patients received a standard anesthetic protocol.	Statistical analysis	This study applied a pharmaceutical care intervention to tonsillectomy.	High	International Journal of Clinical Pharmacy	1.339

						- Quantitative measurements made during the operation					
Hailah Aldosari, Et al. (2020)	Kuwait	Cross-sectional study	All governmental hospitals and polyclinics and private pharmacies in all the five health regions of Kuwait (Capital, Hawalli, Ahmadai, Farwaniyah, and Jahra).	Researchers	Pharmacists working in governmental and private sectors	Data were collected via self-administered questionnaires.	Statistical analysis	Pharmacists' attitudes towards continuing education and to investigate the perceived barriers that hinder pharmacists from being involved in continuing education activities	Fair	Saudi Pharmaceutical Journal	2.879
Salmeen D Babelghaith, Et al. (2020)	Saudi Arabia	Retrospective study	This retrospective study was conducted among inpatients at the Ministry of Health hospital in Jazan, a region in southwestern Saudi Arabia.	Pharmacists	Ten departments: medical, surgical and orthopedic, pediatric, obstetric gynecology, delivery, operation room, anesthesiology, recovery room, adult intensive care, and neonatal intensive care.	Data collected depending on the implementation of Inpatient Pharmacy Intervention Reports File (IPIRF).	Descriptive statistics	The incidence of drug-related problems (DRPs) in different inpatient departments along with the medical team response to pharmacist's action	Good	Journal of Risk Management and Healthcare Policy	0.968
Esra Kucuk, Et al. (2020)	Turkey	Prospective study	This study was conducted at the Hacettepe University Hospital oncology outpatient clinic which	Clinical pharmacists	Oncology department	-On each visit to the clinic during the study period, a clinical pharmacist would review the patients'	Descriptive statistics	The incidence of drug-related problems in patients receiving targeted	Fair	Journal of Oncology Pharmacy Practice	1.85

			provided care to around 80–100 patients per day and ran for five days between 8 am and 4 pm			medical records and ask the patient questions about their health status and drug therapy while they were receiving their treatment infusions.<br>- Identified DRPs were categorized using the Pharmaceutical Care Network Europe (PCNE) v.7 classification system		chemotherapy and/or immunotherapy, and demonstrate the impact of a clinical pharmacist in an outpatient oncology care setting.			
Nesligul Ozdemir. Et al. (2020)	Turkey	Cross-sectional study	This study was carried out in a tertiary care hospital's pediatric bone marrow transplantation unit	Clinical pharmacists	Pediatric bone marrow transplantati on unit	Drug-related problems in this study were identified by using Pharmaceutical Care Network Europe.	Statistical analysis	To demonstrate the importance of clinical pharmacists' participation in the detection, prevention, and management of drug-related problems	Fair	Journal of Clinical and Experimental Health Science	2.644
Lobna S Mostafa, Et al. (2020)	Egypt	Prospective pre–post-interventional study	-The study was conducted at the emergency hospital within Ain Shams University Hospital.<br>- The emergency hospital, which includes a variety of surgical disciplines, is designed for the care of patients who are admitted for surgical purposes without prior appointment, either by	Clinical pharmacists	Emergency sector	-Reviewing medications orders<br>- Categorizing errors according to the National Coordinating Council for Medication Error Reporting and Prevention (NCC MERP).	Statistical analysis	Evaluate the impact of pharmacist-led educational implementations in reducing medication errors	Fair	Journal of Public Health	1.648

			their means or by ambulance.								
Linda Tahaineh, Et al. (2020)	Jordan	Two methodologies: a structured interview technique and a pre-and post-intervention	Obstetrics and gynecology clinics as well as family-planning clinics in public health centers in North Jordan	Clinical pharmacists	Obstetrics and gynecology clinics as well as family-planning clinics	A validated closed- and open-ended questionnaire and educational session	Statistical analysis	Measuring the effect of clinical pharmacist's intervention regarding the risks and benefits associated with the use of combined oral contraceptive (COC) pills.	Fair	Jordan Journal of Pharmaceutical Science	0.270
Sayer I Al-Azzam, Et al. (2016)	Jordan	The study was a non-randomized trial	-At Alsarih Medical Health Center in the north of Jordan.<br>-This health center is a comprehensive governmental healthcare facility that provides primary healthcare services to a population of about 65 000.<br>-Services include emergency care, specialized outpatient clinics in family medicine, internal medicine, pediatrics, dermatology, and obstetrics/gynecology.<br>-The staff at the center comprises 10 physicians and 15 nurses and supporting staff. It services an	Clinical pharmacists	The research interviewed randomly selected patients	Interview	Statistical analysis	Assessing patients' DRPs, proposed appropriate clinical interventions to physicians, and provided appropriate patient counseling to promote adherence and patient safety.	Good	Journal of International Health	1.797

			average of 100 patients per day.								
Shadi Farsaei, Et al. (2014)	Iran	Prospective pre–post-interventional study	The study setting was a 60-bed infectious diseases ward of Imam Khomeini hospital, a multispecialty health care university facility, affiliated to Tehran University of Medical Sciences, Tehran, Iran.	Clinical pharmacists	Infectious diseases ward	- Interventions including insulin, hypoglycemic, and nutritional management were written in the chart of patients by clinical pharmacists.<br>-Then all data were filled into the specific questionnaire.	Statistical analysis	The impact of the clinical pharmacist interventions on the glycemic control	Good	Journal of Infection in Developing Countries	0.703
Iman A Basheti, Et al. (2018)	Jordan	Randomized control trial.	Jordan University Hospital, Amman, Jordan.	Clinical pharmacists	Outpatient clinic	- The clinical pharmacist interviewed patients in both groups at their homes. For patients who refused the home visit, assessments were completed at the clinic.<br>-Questionnaire	Statistical analysis	Identifying treatment-related problems (TRPs) through home visits, assessing type and frequency of TRPs, and eventual effect of resolving TRPs	High	Journal of Pharmaceutical Health Services Research	0.189
Iman A Basheti, Et al. (2016)	Jordan	Randomized control trial.	Jordan University Hospital, Amman, Jordan.	Clinical pharmacists	Outpatient clinic	- The clinical pharmacist interviewed patients in both groups at their homes. For patients who refused the home visit, assessments were completed at the clinic.<br>-Questionnaire	Statistical analysis	Identifying treatment-related problems (TRPs) through home visits, assessing type and frequency of TRPs, and eventual effect of resolving TRPs	High	Journal of Pharma-cotherapy	3.196
Kamel Abdelaziz, Et al. (2015)	Egypt	Prospective study	In a general ICU that contains 22 beds in Riyadh Care Hospital, Saudi Arabia.	Consultants	ICU	Data collected by recording patients' info, DRPs, the cause of admission, and other related information.	Statistical analysis	Identifying and categorizing drug-related problems (DRPs)	Fair	Europe Journal of Hospital Pharmacy	0.892

Amal Akour, Et al. (2017)	Jordan	Randomized control trial.	Women who were visiting community pharmacies or fertility, obstetrics, and gynecology outpatient clinics	Pharmacists	Community pharmacies or fertility, obstetrics, and gynecology outpatient clinics	Booklet and through the questionnaire.	Statistical analysis	The effect of a pharmacist-provided information booklet on increasing the knowledge of Jordanian women about safe and effective OCP use.	High	The European Journal of Contraception and Reproductive health Care	1.382
Ayla M. Tourkmani, Et al. (2018)	Saudi Arabia	Randomized control trial.	-The study was conducted in Al-Wazarat Chronic Diseases Center, a division of the Al-Wazarat Health Care (WHC) Family Medicine Center.<br>-The Chronic Diseases Center consists of 12 specialized clinics, primarily for patients with T2DM, hypertension, dyslipidemia, and bronchial asthma, in addition to a procedures room and support services such as pharmacy, laboratory, and radiology.	Clinical pharmacists and physicians.	The Chronic Diseases Center.	The integrated care program by reviewing the patient charts and arranging appointments with specialties.	Statistical analysis	Assessing the impact of such a program on glycemic control and cardiovascular risk factors.	High	BMC Family Practice	1.332
Razan Adnan Batta, Et al. (2018)	Jordan	Randomized control trial.	Not determined	Clinical pharmacist	Pregnant patients of gestational age 20–28 weeks with diabetes	By using questionnaire and face-to-face interviews	Statistical analysis	Evaluate the role of a clinical pharmacist in the selection of the appropriate medical	High	International Journal of Clinical Pharmacy	1.692

					(type 1, type 2, or GDM)			intervention for patients with hyperglycemia in pregnancy			
Abdulaziz Saleh Almulhim, Et al. (2019)	Saudi Arabia	Retrospective pre-post intervention study	Banner-University Medical Center South is a tertiary care teaching hospital with annual ED visits of 45,000.	pharmacists	Emergency department (ED)	Reviewing the patient's charts, then a structured data collection tool (Excel) was used to perform data collection.	Both descriptive and inferential statistics	Evaluating the performance of our ED pharmacist urine culture follow-up and compare it to the nurse practitioner (NP)-driven follow-up in terms of the appropriateness of chosen antibiotics and, inappropriately treated asymptomatic bacteriuria.	Good	Journal of Pharmaceutical Policy and Practice	0.644
Mohammed Almzeiny, Et al. (2020)	Saudi Arabia	Cross-sectional study	The study was conducted at PSMMC, a 1,350-bed tertiary care referral hospital for members of the Saudi Arabian armed forces and their families. PSMMC is in Riyadh, Saudi Arabia.	pharmacists	The neonatal intensive care unit (NICU), the high dependency unit (HDU), and pediatric nephrology	Questionnaire	Descriptive statistics	Exploring the perceptions and satisfaction of stakeholders (physicians, nurses, and pharmacists) regarding services provided by the patient care area pharmacist (PCAP) initiative.	Fair	Tropical Journal of Pharmaceutical Research	0.504
Adil A. Mahmoud, Et al. (2017)	Saudi Arabia	An interventional, non-randomized,	King Faisal Hospital in Taif City, Kingdom of Saudi Arabia.	pharmacists	Outpatient cardiology clinic	Questionnaire and face to face interview.	Descriptive statistics	Assessing the impact of the practice of PCs on patient outcomes: blood pressure	Fair	International Journal of Pharmaceutical	0.22

		uncontrolled study						control, knowledge of the disease, and adherence to medications.		Science Review and Research	
Majdoleen Al Alawneh, Et al. (2019)	Jordan	Prospective randomized single-blinded intervention-control study	In three main cities in Jordan (Amman, Mafraq, Zarqa), where the majority of the Syrian refugees reside.	Clinical pharmacists	Syrian refugees' clinics in Jordan	Data were collected via 2 home visits.	Statistical analyses	Identifying the type and frequency of TRPs amongst Syrian 92 refugees living in Jordan, and exploring the impact of the HMMR service on reducing the 93 number of the identified TRPs.	Good	Research in Social and Administrative Pharmacy	2.844
Mohamad Ali Hijazi, Et al. (2020)	Lebanon	Cross-sectional study	Community pharmacies	Pharmacists	Community pharmacies	Face to face interview and questionnaire.	Statistical analyses	The role of community pharmacists in weight management	Fair	BMC Health Service Research	0.995
Bushra Hassan Marouf, Et al. (2020)	Iraq	An interventional randomized study.	At the dialysis centers of Kirkuk General Hospital- Directorate of Health in Kirkuk city; a hospital with 250 beds for hospitalization of chronic kidney disease patients.	Clinical pharmacists	Anemic patients on hemodialysis	Reviewing patients' medications and all the interventions	Statistical analyses	The effect of pharmacist interventions in optimizing management of anemia associated with chronic kidney disease	High	Journal of Young Pharmacy	0.180
Rashida Muhammad Umar, Et al. (2020)	Turkey	Prospective study	Marmara University Teaching and Research Hospital Medical Oncology.	Clinical pharmacists	Oncology department	Reviewing patients' health status and medication and using (PCNE) Classification Scheme for Drug-Related Problems V6.2.	Statistical analyses	Intervening in identified drug-related problems.	Good	International Journal of Clinical Pharmacy	1.616

Miri Potlog Shchory, Et al. (2020)	Israel	Quantitative intervention study	Three public hospitals	Researchers	Internal medicine	Questionnaire	Statistical analyses	The effect of an intervention program on the knowledge and attitudes among physicians and nurses regarding ADRs reporting.	Fair	Journal of Pharmacoepdimology and Drug Safety	2.918
MF Najjar, Et al. (2017)	Saudi Arabia	Pre-post interventional study	At King Abdulaziz Medical City in Riyadh, Saudi Arabia.	Clinical pharmacists	Medical wards for Geriatric patients	-Reviewing baseline data about the current prescribing pattern of PIMs for geriatric inpatients and an educational program	Statistical analyses	Reducing the incidence of PIMs among hospitalized geriatric patients.	Good	Journal of Pharmacoepdimology and Drug Safety	2.314
Erdem Topal, Et al. (2020)	Turkey	Cross-sectional study	Three centers located in different parts of Turkey were included.	Researchers	Community pharmacists	Face to face interview and questionnaire	Statistical analyses	Indicating that pharmacists should be included in the training program and be provided continuous training on the use of pMDIs with a spacer device.	Fair	Journal of Asthma	1.899
Fikret Vehbi İZZETTİN, Et al. (2019)	Turkey	Cross-sectional study	Not determined	Clinical pharmacists	Outpatient cardiology clinic.	An interview with the questionnaire.	Statistical analyses	Determining the effectiveness of pharmacist consultation, education, and intervention on each patient’s therapeutic results.	Fair	Journal of Research in Pharmacy	3.90
Elham Alshammari. (2019)	Saudi Arabia	Prospective study	The study was conducted at Prince Sultan Cardiac Center in Riyadh, Kingdom of Saudi Arabia, a 160 full-capacity beds	Clinical pharmacists	Cardiac center	Reviewing and recoded hospitalized patients’ files	Statistical analyses	Clinical pharmacist role in preventing or decreasing drug-related adverse events and	Fair	International Research Journal of Pharmacy	0.765

			instituted for hospitalized patients with cardiac problems or scheduled procedures and also serve outpatient and emergency clinics.					eventually decrease hospitalization and costs			
Kinda T Alkoudsi et al. (2020)	Jordan	Randomized control trial	Females diagnosed with PCOS, living in the national capital cities of Syria (Damascus) and Jordan (Amman), were approached while visiting community pharmacies for study recruitment.	Clinical pharmacist	Females diagnosed with PCOS	-The database was collected from patients and by using a questionnaire.	Statistical analysis	The impact of a pharmaceutical care service for PCOS on participants' anxiety and depression scores and severity.	High	Research in Social and administrative Pharmacy	2.844
Shaymaa Abdalwahed Abdulameer. (2018)	Iraq	Cross-sectional study	Three areas from two large distract zone named Al-Kharkh (west of the Tigris: four distract zone) and Al-Rusafa (east of the Tigris: six distract zones).	Pharmacist	Community pharmacies	Data collected through a face-to-face interview.	Statistical analysis	Assess the diabetes knowledge and pharmaceutical care practice among registered and unregistered pharmacists	Fair	Journal of Applied Pharmaceutical Science	0.252
Amr S. Shalaby. (2017)	Egypt	Cross-sectional study	The study was conducted in Menoufia Governorate, which is located in the Nile Delta, in the northern part of the country, and to the north of Cairo. It consists of nine administrative centers that include 10 cities.	Health care professional	Department of Neuropsychiatry	Data collected through a questionnaire.	Statistical analysis	Exploring the attitude of a sample of community pharmacists and their assistants toward psychiatric patients	Fair	Journal of Middle East Current Psychiatry	0.15

Khizra Sultana. Et al. (2016)	Saudi Arabia	Cross-sectional study	The Pharmaceutical department is divided into outpatient, inpatient, and clinical pharmacy services. In Riyadh, we have 33 clinical pharmacists and 175 pharmacists involved in outpatient, inpatient, and discharge counseling services.	Pharmacists	Saudi Pharmacists.	Data collected through a questionnaire.	Descriptive analysis	Assessing the attitude, motivators, and barriers of pharmacists to take part in PBR.	Fair	Journal of Pharmaceutical Policy and Practice	0.292
Noor Alsalimy, Et al. (2017)	Qatar	Retrospective	Rumailah Hospital (RH) is a tertiary public hospital in Qatar that provides several healthcare services for sub-acute and long-term care patients, including surgical services for ear, nose, and throat (ENT), ophthalmology, and plastic surgery.	Pharmacists	All ADR reports from all RH units.	A paper-based reporting form.	Descriptive and inferential statistics	Reviewing ADR reports	Fair	Journal of Drugs and Therapy perspective	0.21
Ramzi Shawahna. (2020)	Palestine	Cross-sectional study	Healthcare facilities.	Pharmacists	Patients admitted to or visiting healthcare facilities	Questionnaire	Statistical analysis	Developing and achieving consensus on what activities and services to use as KPIs to capture and measuring the impact of pharmacists in	Fair	Evidence-Based Complementary and Alterative Medicine	2.64

								integrative healthcare facilities.			
Hani MJ Khojah. (2019)	Saudi Arabia	Cross-sectional study	Private community pharmacies (353 pharmacies) and their locations in Madinah	Pharmacists	Private community pharmacies	Questionnaire	Descriptive analysis	Investigating the level of provision of customer education regarding the effects of sedating antihistamines on driving skills by private community pharmacies	Fair	Journal of International Medical Research	1.287
Anan S Jarab Et al. (2020)	Jordan	Cross-sectional study	Queen Alia Heart Institute at Royal Medical Services (RMS). This clinic is considered as one of the largest hospitals in Jordan that provides healthcare services to more than 100 angina patients daily. All patients who had IHD for at least 1 year	Clinical pharmacists	Outpatients cardiac clinic	Questionnaire	Descriptive analysis	Evaluating HRQOL and to explore the factors associated with poor HRQOL among patients with angina	Good	Quality of Life Research Journal	2.392
Rania E. Ghanem, Et al. (2020)	Palestine	Cross-sectional study	Community pharmacies located in cities, villages, and refugee camps in different governorates were visited.	Pharmacists	Community pharmacies	Questionnaire	Descriptive analysis	Exploring their knowledge, attitude, and practice for acne management.	Fair	The International Journal of Dermatology	1.794

Nour Makkaoui, Et al. (2020)	Lebanon	Cross-sectional study	Licensed community pharmacies in Lebanon	Pharmacists	Community pharmacies	Data was collected through an interview and questionnaire	Statistical analysis	Exploring the knowledge, attitude, and practice that community pharmacists of Lebanon hold concerning checking for drug interactions.	Fair	Journal of Public Health	1.648
Anan S. Jarab, Et al. (2020)	Jordan	Cross-sectional study	Queen Alia Heart Institute in the Royal Medical Services. This clinic is considered as one of the largest hospitals in Jordan that provides healthcare services to more than 100 angina patients daily.	Pharmacists	Outpatients cardiac clinic	Questionnaire	Descriptive analysis	Evaluating medication adherence and exploring the factors associated with medications non-adherence among patients with angina	Fair	Jordan Journal of Pharmaceutical Science	0.270
Dhfer Mahdi AlShayban, Et al. (2020)	Saudi Arabia	Cross-sectional study	Hospital pharmacies of King Fahd Hospital of the University located in the city of Khobar and, Al Zahra Hospital in Qatif city, as well as community pharmacies located in cities of Khobar, Dammam, and Qatif served as venues.	Pharmacists	Hospital and community pharmacies.	Questionnaire	Descriptive analysis	Evaluating patients' satisfaction and WTP for pharmacist counseling	Fair	Journal of Frontiers in Pharmacology	4.225

Kholoud Dweib, Et al. (2020)	Palestine	Cross-sectional study	MOH primary health-care clinics of Bethlehem and North Hebron	Pharmacists	Kidney transplant clinic	Questionnaire	Descriptive analysis	Measuring QoL among renal transplant patients	Fair	Saudi Journal of Kidney Disease and Transplantation	0.72
F I Al-Saikhan. (2020)	Saudi Arabia	Prospective, Cross-sectional study	Prince Sattam Bin Abdulaziz University Hospital in Alkharj.	Clinical pharmacist	Outpatient anticoagulation clinic	Questionnaire	Descriptive analysis	Examining the association between WTA and HRQOL in patients being treated with warfarin in Saudi Arabia.	Fair	Nigerian Journal of Clinical Practice	0.634

Günay Arsoy, Et al. (2018)	Cyprus	Cross-sectional study	The included only experienced community pharmacists with 5 or more years' experience as stipulated by Turkish and Northern Cyprus Pharmacists' Association's norms.	Trained pharmacists	Community pharmacies	-The database was collected from patients and by face-to-face interview and questionnaire	Statistical analysis	Evaluating the effectiveness of the pharmacist-led educational intervention for improving patients' QoL and perceived symptom severity.	Good	Medicina Journal	2.861
Maram Gamal Katoue. (2020)	Kuwait	Cross-sectional study	Kuwait's governmental healthcare system is organized into three levels of healthcare delivery: they are primary, secondary, and tertiary. Primary healthcare is delivered through healthcare centers distributed across the different areas of the State. Secondary healthcare is provided	Pharmacist	Governmental hospital	Data were collected from participants using focus group interviews	Thematic analysis	Exploring the perceptions of hospital pharmacists about the enablers and challenges to expanding their scope of practice to include direct patient care.	Fair	Journal of Evaluation in Clinical Practice	1.684

			through six general hospitals, while tertiary healthcare is delivered through many specialized hospitals and health centers.								
Elif Ertuna, Et al. (2019)	Turkey	Retrospective	In the acute geriatric ward (10 beds) of a government-run 1,800-bed tertiary university hospital in Turkey.	Clinical pharmacists	Geriatric ward	Data were collected by reviewing medication reports	Statistical analysis	Defining and classifying DRPs, pharmacist interventions, and frequently prescribed medications concerning possible DRPs in patients admitted to the geriatric ward.	Fair	Journal of Clinical Intervention in Aging	1.0
Afsaneh Vazin, Et al. (2018)	Iran	Prospective	Namazi Hospital, affiliated to Shiraz University of Medical Sciences (Shiraz, Iran) is a general tertiary referral center with 50 wards and nearly 1000 beds.	Trained pharmacist and Clinical pharmacists	Clinical wards of Namazi Hospital	Data collected from the Hospital Information System and patient records.	Statistical analysis	Evaluating the impact of an intervention by the pharmaceutical care unit on the use pattern of high-value medications and their direct costs in a referral hospital.	Fair	BMC Health Service Research Journal	1.057
Mostafa A Sayed Ali, Et al. (2018)	Egypt	Prospective	Assiut University Hospital. The inpatient wards have a capacity of 48 beds and the ICU unit comprises 18 beds.	Clinical pharmacists	In-patient wards and intensive care unit of the Department of Neurology	By reviewing all patients' drug charts.	Statistical analysis and descriptive analysis	Identifying common DRPs in neurology and document clinical pharmacists' medication-related interventions.	Fair	International Journal of Clinical Pharmacy	1.692
Reem A Bahmaid, Et al. (2018)	Saudi Arabia	pre-test/post-test quasi-experimental	Not determined	Trained research coordinator	Three tertiary care settings: King Fahad Medical City,	Data were collected by using a questionnaire	Statistical analysis	Assessing the impact of research educational intervention on	Fair	Cureus	1.15

		interventional study			King Faisal Specialist Hospital and Research Center, and Prince Sultan Cardiac Center			knowledge, attitudes, perceptions, and pharmacy practices towards evidence-based medicine			
Pallivalapilla Abdul Rouf, Et al. (2018)	Qatar	Cross-sectional study	Not determined	Pharmacists	Hospitals, community pharmacies	Data were collected by using a questionnaire	Descriptive analysis	Determining the knowledge and practice characteristics of pharmacists in Qatar about medicines use in pregnancy.	Fair	Estran Mediterranean Health Journal	0.694
Abdikarim Mohamed Abdi, Et al. (2018)	Cyprus	Prospective	Community pharmacists working in pharmacies in North Cyprus. There was 190 licensed pharmacies majority operating in the 5 main cities in North Cyprus	Clinical pharmacists	Community pharmacies	Data were collected by using a questionnaire	Statistical analysis	Assessing community pharmacist's perceptions and practice of pharmaceutical care	Fair	Journal of Pharmaceutical Research International	0.07
Ata Mahmoodpoor, Et al. (2018)	Iran	Cross-sectional study	Shohada University Hospital.<br>Not determined	Clinical pharmacists	ICU	Questionnaire	Descriptive statistical analysis	Assessing clinical pharmacy services in the intensive care unit (ICU)	Good	Journal of Research in Pharmacy Practice	0.00
Muath Fahmi Najjar, Et al. (2018)	Saudi Arabia	Prospective and Pre-Post interventional study.	Department of Medicine at King Abdulaziz Medical City.<br>Not determined	Clinical pharmacists	Medical wards<br>(Hospitalized geriatric patients)	Data was reviewed baseline data about the current prescribing pattern of PIMs for geriatric inpatients data obtained from the	Statistical analysis	Assessing the effectiveness of a combined intervention program comprising an educational and clinical pharmacist intervention to reduce the incidence of PIMs	Good	Therapeutic and Clinical Risk Management	0.725

						Hospital Information System		among hospitalized geriatric patients.			
Shaymaa Abdalwahed Abdulameer. (2018)	Iraq	Cross-sectional study	Baghdad city has two large district areas named Al-Karkh and Al-Rusafa. The random cluster sampling method was used to select three areas from each.	Pharmacist	Community pharmacies	Data were collected by using a questionnaire	Statistical analysis	Assessing the pharmaceutical care practice among registered pharmacists.	Fair	International Journal of COPD	3.157
Huda G El-Refae, Et al. (2017)	Jordan	Prospective	The University of Jordan Hospital.<br><br>Not determined	Clinical pharmacists	Cardiology ward	By telephone call or hospital visit for a medication refill.	Statistical analysis	Assessing physician acceptance of clinical pharmacist suggestions; and determining the impact on costs to patients.	Good	Tropical Journal of Pharmaceutical Research	0.48
Haider Al-Baghdadi, Et al. (2017)	Cyprus	Prospective	Near East University Hospital, which is the largest and leading medical facility in Nicosia, Northern Cyprus. The services of the hospital are performed within a compound comprising over 56 thousand square meters of indoor space including 209 private, single-patient rooms, 8-operating theatres, 30-bed Intensive Care Unit, 17-bed	Clinical pharmacists	Cardiology and cardiovascular surgery departments	By reviewing patients' medications using PCNE to identify DRPs	Statistical analysis	Assessing the implementation of ward-based clinical pharmacy services in cardiovascular clinics and describing the prevalence and nature of encountered DRPs with the associated factors.	Fair	International Journal of Clinical Pharmacy	1.508

			Neonatal Intensive Care Unit, and advanced laboratory.								
Ziyad Alrabiah, Et al. (2017)	Saudi Arabia	Prospective cross-sectional study	Not determined	Clinical pharmacists	Community pharmacists	Data were collected by using a questionnaire	Both descriptive and analytic statistics	Evaluating knowledge of CPs about medication safety during pregnant	Fair	Saudi Pharmaceutical Journal	3.11
Rana Abu Farha, Et al. (2017)	Jordan	Cross-sectional study	Jordan University Hospital.<br><br>Not determined	Clinical pharmacists	Cardiac outpatient clinic	Data were collected by using a questionnaire	Both descriptive and statistical analysis	Identifying the influence of different types of DRPs on the HRQOL of hypertensive patients.	Fair	Journal of Pharmacy Practice	0.451
Turki Assiri, Et al. (2017)	Saudi Arabia	Prospective	-At Prince Sultan Cardiac Center (PSCC).<br><br>- PSCC is a specialized healthcare center that provides comprehensive cardiovascular services to all armed forces personnel, their dependents, and other patients referred to the center for further evaluation and specialized treatment and includes about 190 beds dedicated	Pharmacists	Cardiac center	Data were collected by reviewing the patient's records, medical and medication history, diagnosis, and discharge (treatment) plan.	Descriptive analysis	Determine the impact of pharmacist's counseling and review of discharge prescriptions for patients discharged from a specialized cardiac center	Fair	Tropical Journal of Pharmaceutical Research	0.444

			to adult and pediatric cardiac patients.								
Aida Sefidani Forough, Et al. (2017)	Iran	Prospective	Amiral Momenin General Hospital	Pharmacists	Elderly diabetic patients (visited the hospital's outpatient department	Data were collected through interviews using researcher-administered questionnaires as well as patients' medical records.	Descriptive statistical analysis	Evaluating the impact of pharmacist-conducted educational intervention on reducing errors related to inappropriate insulin pen use.	Fair	Journal of Research in Pharmacy Practice	0.00
Ali A. ALMITWAZI, Et al. (2017)	Saudi Arabia	Retrospective	At Prince Sultan Cardiac Center (PSCC).<br>- PSCC is a specialized healthcare center that provides comprehensive cardiovascular services to all armed forces personnel, their dependents, and other patients referred to the center for further evaluation and specialized treatment and includes about 190 beds dedicated to adult and pediatric cardiac patients.	Clinical pharmacists	Cardiac center	Data were extracted from the hospital's electronic documentation.	Descriptive statistical analysis	Assessing the clinical pharmacist interventions and probable impact of these interventions in cost minimization by reducing direct medication costs at a specialized cardiac center.	Fair	Latin American Journal of Pharmacy	0.401

Nibal R Chamoun, Et al. (2016)	Lebanon	Retrospective	At a teaching hospital	Clinical pharmacists	Cardiology and infectious diseases departments	By reviewing patients’ documentation and reporting sheets	Descriptive statistical analysis	Showing the impact of pharmacy interventions in reducing MRPs	Fair	International Journal of Clinical Pharmacy	1.555
Khawla Abu Hammour, Et al. (2016)	Jordan	Cross-sectional study	At four tertiary hospitals (two private and two teaching hospitals)	Pharmacists	Two private and two teaching hospitals	Data was collected by using a questionnaire	Descriptive analysis	Gaining an insight into hospital pharmacists' current practice and perceptions towards medicine reconciliation and identifying common challenges	Fair	Journal of Evaluation in Clinical Practice	1.25
Farzaneh Foroughinia, Et al. (2016)	Iran	Prospective cross-sectional study	Shahid Faghighi Hospital	Clinical pharmacists	Neurology ward	Checked the patients' files and pharmacotherapy sheets and categorized DRPs using PCNE.	Descriptive analysis	The role of the clinical pharmacist in the detection and prevention of these drug problems and evaluated the frequency and type of DRPs	Fair	Journal of Research in Pharmacy Practice	0.00
Laika Gökçekuş, Et al. (2016)	Cyprus	Prospective	Four community pharmacies in North Cyprus.	Clinical pharmacists	Community pharmacies	Data was collected by using form and PCNE classification.	Statistical analysis	Investigate the role of the community pharmacist in identifying, preventing, and resolving drug-related problems (DRPs)	Good	Tropical Journal of Pharmaceutical Research	0.48

Hani Abdelaziz, Et al. (2016)	Qatar	Retrospective	Hamad General Hospital.<br><br>Not determined	Clinical pharmacists	Emergency department	Data were collected by reviewing patient profiles and an electronic system was used to record the interventions recommended	Descriptive analysis	Characterizing the contributions of clinical pharmacists in a short stay unit of ED to implement and scale-up the service to all ED areas in the future.	Fair	International Journal in Clinical Pharmacy	1.555
Maguy Saffouh El Hajj, Et al. (2016)	Qatar	Cross-sectional study	Public and private ambulatory care clinics, in outpatient hospital pharmacies, and community pharmacies	Pharmacists	Public and private ambulatory care clinics, in outpatient hospital pharmacies, and community pharmacies	Data was collected by using a questionnaire	Statistical analysis	Describing pharmacists' attitudes towards their involvement in CVD prevention and to assess their perceived barriers to the provision of CVD prevention services	Fair	International Journal in Clinical Pharmacy	1.555
Amal K. Suleiman, Et al. (2016)	Saudi Arabia	Cross-sectional study	Not determined	Pharmacists	Community pharmacists	Data was collected by using a questionnaire	Descriptive analysis	Exploring pharmacists' attitudes regarding pharmaceutical care in chronic kidney disease (CKD) and end-stage renal disease (ESRD) patients.	Fair	Archives of Pharmacy Practice	0.252
Maguy Saffouh El Hajj, Et al. (2016)	Qatar	Cross-sectional study	Community Pharmacies	Clinical Pharmacists	Community Pharmacies	Data was collected by using a questionnaire	Statistical analysis	Examining the extent of pharmaceutical care practice and the barriers to pharmaceutical care provision	Good	Journal of Evaluation in Clinical Practice	1.681

Gholamhossein Mehralian, Et al. (2015)	Iran	Cross-sectional study	Community Pharmacies	Pharmacists	Community Pharmacies	Data was collected by using a questionnaire	Descriptive analysis	Evaluating pharmacists' practice and attitude toward pharmaceutical care (PhC) and present barriers	Fair	Journal of Pharmaceutical Health services Research	0.270
Niaz Al-Somai, Et al. (2014)	Saudi Arabia	Retrospective	The hospital is a tertiary center that provides CCU, CSICU, Cardiac, Hematology, ICU, Medical, Neuroscience, Oncology, and specialized surgery services.	Clinical pharmacists	CCU, CSICU, Cardiac, Hematology, ICU, Medical, Neuroscience, Oncology, and specialized surgery services	Data were collected by reviewing the medical chart, lab tests, and culture reports	Statistical analysis	Evaluating the role of clinical pharmacist and ID interventions in optimizing antimicrobial therapy.	Fair	Saudi Pharmaceutical Journal	1
Richard Hooper, Et al. (2014)	Qatar	Prospective	The study was conducted in four primary health care clinics	Pharmacists	Primary health care clinics	Data were collected by reviewing documented information of patients and by using PCNE classification.	Statistical analysis	Characterizing and analyze interventions documented by pharmacists in outpatient pharmacies	Fair	International Journal Clinical Pharmacy	1.616
Dalal M. Al-Taweel, Et al. (2013)	Kuwait	Cross-sectional study	Community pharmacies	Pharmacists	Community pharmacies	Data were collected using a questionnaire and an interview.	Descriptive and thematic analysis	Identifying pharmacists' potential contributions to the delivery of pharmaceutical care to patients with type 2 diabetes	Fair	International Journal of Diabetes in Developing Countries	0.525

Salah M AbuRuz, Et al. (2013)	Jordan	Pre-Post intervention	At the nephrology wards (10 beds) of one of the largest general teaching hospitals in Jordan.	Clinical pharmacists	Nephrology wards	Data were screened patients' medical files	Statistical analysis	Implementing and evaluating the impact of pharmaceutical care service for hospitalized chronic kidney disease (CKD) patients	Good	International Journal of Clinical Pharmacy	1.25
Mohammed S. Alsultan, Et al. (2013)	Saudi Arabia	Cross-sectional study	Hospital pharmacy	Clinical pharmacists	Hospital pharmacy	Data were collected by a questionnaire	Descriptive statistical	Describing and characterize the trends in pharmacist's monitoring of medication therapy, and describe the monitoring activities of pharmacists.	Fire	Saudi Pharmaceutical Journal	1
Hossein Khalili, Et al. (2013)	Iran	Prospective	At 60-bed infectious diseases ward of Imam Hospital, a multispecialty health care university setting in Tehran, Iran.	Clinical pharmacists	Infectious diseases ward	Data were collected by a questionnaire and medical charts of the patients	Descriptive statistical analysis.	Impact of clinical pharmacy interventions on decreasing direct medication costs	Fair	European Journal of Internal Medicine	3.66
Maram G Katoue, Et al. (2013)	Kuwait	Cross-sectional study	Community pharmacies	Pharmacists	Community pharmacies	Data were collected via a face-to-face structured interview of the pharmacists using a pre-tested questionnaire.	Descriptive and comparative analysis	The role of community pharmacists in the care of patients with metabolic syndrome.	Fair	International Journal of Clinical Pharmacy	1.25

Nadir Yalçına, Et al. (2019)	Turkey	Prospective study	University hospital.<br><br>Not determined	Clinical pharmacist	Psychiatric ward	The data was collected by using a specific scale and an interview.	Statistical analysis	Evaluating whether drug education during the discharging period affects compliance.	Fair	International Clinical Psycho-pharmacology	3.098
Emre KARA, Et al. (2019)	Turkey	A prospective, cross-sectional study	At the Infectious Diseases Outpatient Clinic of the Hacettepe University Adult Hospital.<br><br>Not determined	Clinical pharmacist and physician	Infectious Diseases Outpatient Clinic	A face-to-face interview with a clinical pharmacist. PCNE Classification V 7.0 was used to classify incident drug-related problems (DRPs).	Statistical analysis	Determining DRPs and to evaluate clinical pharmacist interventions among PLWHA	Fair	Turkish Journal of Medical Science	0.717
Ceylan Paksoy, Et al. (2018)	Turkey	Prospective study	At the oncology outpatient clinic located in Istanbul.<br><br>Not determined	Clinical pharmacist	Oncology outpatient clinic	Data was obtained from the patient’s records and medication.	Statistical analysis	Evaluating potentially inappropriate medication use in elderly patients with cancer.	Fair	Journal of Oncology Pharmacy Practice	1.85
Kamer TECEN-YUCEL, Et al. (2018)	Turkey	A descriptive, cross-sectional, prospective study	At the medical oncology outpatient clinic of a University Oncology Hospital. The clinic is run by two consultant doctors	Clinical pharmacist	Oncology outpatient clinic	Data was obtained from the patient’s records.	Descriptive analysis and statistical analysis	To identify and evaluate drug-drug interactions and adverse effects related to TKIs in patients	Fair	International Journal of Hematology and Oncology	1.992

Fikret V. Izzettin, Et al. (2017)	Turkey	Prospective study	At the oncology unit in one of the Teaching and Research Hospital in Istanbul.<br><br>Not determined	Clinical pharmacist	Oncology unit	Data were collected from the medical records of the patients and face- to – face interview.	Statistical analysis	Assessing the occurrence of drug-related problems and the importance of the effective provision of patient education and appropriate recommendations	Fair	Marmara Pharmaceutical Journal	0.31
Songül Tezcan, Et al. (2016)	Turkey	Prospective study	At the outpatient chemotherapy unit of Marmara University Pendik Training and Research Hospital	Clinical pharmacists	Outpatient chemotherapy unit	Data was obtained from the patient's records and an interview.	Statistical analysis	Determining and evaluate the pharmaceutical care needs and quality of life of patients with colorectal cancer.	Fair	Europe Journal of Hospital Pharmacy	0.538
SuleApikoglu-Rabus, Et al. (2016)	Turkey	Prospective study	Not determined	Pharmacists	Pharmacy	Data were collected by using a questionnaire, patients records, and a face-to-face interview	Statistical analysis	Describing drug-related problems in patients with asthma and COPD	Fair	Journal of Respiratory Medicine	3.237
A. Selcuk, Et al. (2015)	Turkey	Retrospective study	Not determined	Clinical pharmacists	Internal medicine	Data was obtained from the patient's records.	Statistical analysis	The impact of services, including medication reconciliation and medication review, on the hospital admission process for elderly patients	Fair	Pharmazic	1.37

Abdelrahman G . Tawfk, Et al. (2020)	Egypt	Simulated client random study	Community pharmacies	Pharmacists	Community pharmacies	Data obtained from the interviews	Descriptive analysis	Investigate the quality of community pharmacies management of self-medication requests of tetracycline for pregnant women.	Good	International Journal of Clinical Pharmacy	1.616
Bashayr Alsuwayni, Et al. (2020)	Saudi Arabia	Prospective study	At an academic hospital.<br><br>Not determined	Clinical pharmacists	Diabetes clinic	Data was collected by using patient electronic health records.	Descriptive analysis	Evaluating diabetes-related health outcomes in a pharmacist-led diabetes clinic in terms of HgbA1C level, guideline-recommended routine screenings, medication adherence, and biomarkers of other comorbidities.	Fair	Saudi Pharmaceutical Journal	2.879
Abdel-Hameed I. Ebid, Et al. (2020)	Egypt	Prospective study	Ahmed Maher Teaching Hospital, in Cairo, Egypt. The outpatient clinic provides usual care services daily with regular follow up clinic visits every 1 to 3months,	Clinical pharmacists	Diabetes outpatient clinics.	Data was collected for each patient through an interview, lab reading, and hospital records.	Descriptive analysis and statistical analysis	Investigating the impact of the clinical pharmacist interventions on glycemic control and other health-related clinical outcomes in patients with type 2 diabetes	Good	Hospital Pharmacy Sage Journal	0.55
